# Hydroxamate Production as a High Affinity Iron Acquisition Mechanism in *Paracoccidioides* Spp

**DOI:** 10.1371/journal.pone.0105805

**Published:** 2014-08-26

**Authors:** Mirelle Garcia Silva-Bailão, Elisa Flávia Luiz Cardoso Bailão, Beatrix Elisabeth Lechner, Gregory M. Gauthier, Herbert Lindner, Alexandre Melo Bailão, Hubertus Haas, Célia Maria de Almeida Soares

**Affiliations:** 1 Laboratório de Biologia Molecular, Instituto de Ciências Biológicas, Universidade Federal de Goiás, Goiânia, Goiás, Brazil; 2 Programa de Pós-Graduação em Patologia Molecular, Universidade de Brasília, Brasília, Brazil; 3 Unidade Universitária de Iporá, Universidade Estadual de Goiás, Iporá, Goiás, Brazil; 4 Division of Molecular Biology/Biocenter, Innsbruck Medical University, Innsbruck, Austria; 5 Division of Clinical Biochemistry/Biocenter, Innsbruck Medical University, Innsbruck, Austria; 6 Department of Medicine, Section of Infectious Diseases, University of Wisconsin, Madison, Wisconsin, United States of America; East Carolina University School of Medicine, United States of America

## Abstract

Iron is a micronutrient required by almost all living organisms, including fungi. Although this metal is abundant, its bioavailability is low either in aerobic environments or within mammalian hosts. As a consequence, pathogenic microorganisms evolved high affinity iron acquisition mechanisms which include the production and uptake of siderophores. Here we investigated the utilization of these molecules by species of the *Paracoccidioides* genus, the causative agents of a systemic mycosis. It was demonstrated that iron starvation induces the expression of *Paracoccidioides* ortholog genes for siderophore biosynthesis and transport. Reversed-phase HPLC analysis revealed that the fungus produces and secretes coprogen B, which generates dimerumic acid as a breakdown product. Ferricrocin and ferrichrome C were detected in *Paracoccidioides* as the intracellular produced siderophores. Moreover, the fungus is also able to grow in presence of siderophores as the only iron sources, demonstrating that beyond producing, *Paracoccidioides* is also able to utilize siderophores for growth, including the xenosiderophore ferrioxamine. Exposure to exogenous ferrioxamine and dimerumic acid increased fungus survival during co-cultivation with macrophages indicating that these molecules play a role during host-pathogen interaction. Furthermore, cross-feeding experiments revealed that *Paracoccidioides* siderophores promotes growth of *Aspergillus nidulans* strain unable to produce these iron chelators. Together, these data denote that synthesis and utilization of siderophores is a mechanism used by *Paracoccidioides* to surpass iron limitation. As iron paucity is found within the host, siderophore production may be related to fungus pathogenicity.

## Introduction

The requirement of iron for growth and proliferation is a feature of virtually all organisms, with the exception of a few bacteria [1], [2]. The biological significance of iron lies on its ability to cycle between two oxidation states: the reduced ferrous (Fe^2+^) and oxidized ferric (Fe^3+^). The capacity to accept and donate electrons gives iron a redox versatility to function as a cofactor for various cellular enzymes involved in several essential biological processes including respiration, the tricarboxylic acid cycle, synthesis of amino acids, deoxyribonucleotides, lipids and sterols as well as oxidative stress detoxification [1]. Although essential, iron can also be toxic in high concentrations since Fe^2+^ has the potential to generate cell damaging reactive oxygen species (ROS) via the Fenton/Haber Weiss reaction [3], [4]. Thereby, cellular iron homeostasis depends on the precise regulation of iron acquisition, utilization and storage.

Under aerobic conditions, iron is oxidized and Fe^3+^ is essentially insoluble in water at neutral pH [5]. Beyond the environmental low iron availability, pathogenic microorganisms are also confronted by iron scarcity during interaction with the host. In mammalian hosts, the assimilated iron is bound to proteins, such as hemoglobin, transferrin, ferritin and lactoferrin [6]. Following infection, iron concentrations in extracellular fluid and plasma decrease [7]. Macrophages play an important role in the iron withholding. These defense cells limit the release of iron obtained from damaged and senescent erythrocytes and, under the influence of cytokines, inhibit multiplication of phagocytosed microorganisms by moving iron from the phagosome to cytoplasmic ferritin [8], [9]. Since both host and pathogen require iron for metabolism, the control over access to this nutrient can dictate the fate of an infection.

Microorganisms, including fungi, have evolved high affinity uptake strategies for iron acquisition in order to overcome the low bioavailability of this ion in aqueous environments (concentration of free Fe^3+^ approximately 10^−18^ M at pH 7) and within mammalian hosts (concentration of free iron in serum in the order of 10^−24^ M) [10]. One of these strategies consists of the synthesis and secretion of siderophores, defined as low molecular weight organic chelators with high affinity for Fe^3+^ [11]. Such molecules are produced under iron limiting conditions and make insoluble Fe^3+^ available for consumption [11]. The high affinity for iron allows siderophores to compete with host proteins transferrin and lactoferrin. Indeed, the pathogen *Aspergillus fumigatus* overcomes the iron limitation of serum by secreting siderophores which remove iron from serum transferrin [12], [13].

With the exception of carboxylates produced by zygomycetes [14], virtually all fungal siderophores are hydroxamates, derived from the non proteinogenic amino acid ornithine [15] (**Figure S1**). In the proposed biosynthetic pathway for fungal hydroxamates, ornithine-N^5^-monooxygenase (SidA) catalyzes N^5^-hydroxylation of ornithine [13], [15], [16]. The hydroxamate group is formed next by N^5^-acylation of N^5^-hydroxyornithine catalyzed by N^5^-transacylases [15]. In *A. fumigatus* two transacylases, which add different acyl groups to hydroxyornithine, were identified thus far: SidF [17], which adds anhydromevalonyl-CoA, and SidL [18], which catalyzes the addition of acetyl-CoA. In this step, the pathway for distinct siderophores splits for the first time since the choice of the acyl group defines the nature of the molecules. Ferricrocin and ferrichrome, for example, are linked to acetyl whilst fusarinines and coprogens possess anhydromevalonyl [19]. The latter moiety is derived of mevalonate, from the ergosterol biosynthetic pathway, by the subsequent action of the acyl-CoA ligase SidI and the enoyl-CoA hydratase SidH [20]. The following step is the covalent linkage of hydroxamates via peptide (ferrichromes, coprogens) or ester bonds (fusarinines, coprogens), performed by non-ribosomal peptide synthetases (NRPSs). The NRPSs SidD and SidC are involved, respectively, in the synthesis of extra- and intracellular siderophores in *A. fumigatus* [17]. Given the role of siderophores as iron scavengers, the ability to produce these molecules is advantageous for pathogenic microorganisms and has been considered a virulence attribute for either human or plant fungal pathogens [17], [21], [22], [23], [24], [25].

The dimorphic fungal pathogens of *Paracoccidioides* genus belong to two species, *Paracoccidioides brasiliensis* and *Paracoccidioides lutzii*. These fungi are the causative agents of paracoccidioidomycosis, a systemic mycosis endemic in Latin America. *P. brasiliensis* includes four cryptic species, S1, PS2, PS3 [26], [27] and PS4 [28], [29], each containing a different number of isolates. *P. lutzii* comprises the previously described “*Pb*01-like” group [30], [31]. Once inhaled by the host, fungal propagules are converted into yeasts in the lungs, from where they can disseminate throughout the body. It was already demonstrated that both mycelial and yeasts forms of *P. brasiliensis* have a metabolic requirement for iron [32] and that iron availability increases the susceptibility of mice to fungus infection [33]. A former report described that *P. brasiliensis* infected patients who have restricted pulmonary disease exhibit no alterations in transferrin saturation or in levels of serum iron. On the other hand, low iron concentrations and reduced saturation of transferrin were found in patients with disseminated disease [34], [35]. Differential gene expression analysis revealed that genes involved in high-affinity iron uptake were induced in *P. lutzii* upon infection of mice and during the incubation with human blood and plasma [36], . It was also demonstrated recently that the human plasma protein hemopexin, which tightly binds to heme group, associates with *P.brasiliensis* cell wall [38].

Taken together, these data demonstrate that the fungus faces iron deprivation within the host and have to overcome the scarcity of this micronutrient. Even though the production of iron chelants by *Paracoccidioides* was already reported, the details about this iron acquisition pathway as well as the nature of the produced molecules were unknown [32]. In a previous study, we demonstrated that *P. brasiliensis* and *P. lutzii* genomes encode orthologs for siderophore biosynthesis (*sidA*, *sidF*, *sidC*, *sidD*) as well as siderophore uptake genes (*sit1*, *mirB*, *mirC*) [39]. In the current study we show that iron limiting conditions trigger synthesis and secretion of hydroxamates coprogen B and dimerumic acid by *Paracoccidioides* genus. The fungus also produces ferricrocin and ferrichrome C as intracellular siderophores. Additionally, both *P. brasiliensis* and *P. lutzii* are able to grow in presence of siderophores as irons sources, including the xenosiderophore ferrioxamine. Siderophore utilization is also important during fungal infection as demonstrated by interaction with macrophages. The findings point to a possible role of siderophores in fungal pathogenicity.

## Materials and Methods

### Strains and growth conditions

Fungal strains used in this study are listed in **Table 1**. *Paracoccidioides* yeasts cells were maintained in brain heart infusion (BHI) medium supplemented with 4% glucose at 36°C. Except for expression analysis under infectious conditions, all the experiments were performed with strains cultivated in chemically defined medium MMcM [40] after growth to exponential phase in liquid BHI and two washes with phosphate buffered saline solution 1X (PBS 1X; 1.4 mM KH_2_PO_4_, 8 mM Na_2_HPO_4_, 140 mM NaCl, 2.7 mM KCl; pH 7.4). For growth on iron sources, *Pb*01 and *Pb*18 were incubated in MMcM with no iron addition and containing 50 µM of bathophenanthroline-disulfonic acid (BPS; B-1375 Sigma-Aldrich, St. Louis, MO), a ferrous iron-specific chelator, for 24 h under rotation. Cells were collected by centrifugation and washed twice with PBS 1X. Serial 10-fold dilutions of cellular suspensions were then spotted on MMcM agar plates containing 50 µM BPS supplemented or not with 10 µM of the siderophores dimerumic acid (DA), ferricrocin (FC) and ferrioxamine (FO). Agar plates supplemented with 10 µM ammonium ferrous sulfate and ammonium ferric citrate, in the absence of BPS, were used as controls. DA and FC were purchased from EMC Microcollections, Tuebingen, Germany. FO was prepared by incubating equal molar amounts of FeCl_3_ and deferoxamine mesylate (D9533 Sigma-Aldrich, St. Louis, MO) together in 1 M Tris pH 7.4 for 30 min at room temperature.

**Table 1:** *Paracoccidioides* strains used in this study.

Species (isolate)	Cryptic species	Reference
*Paracoccidioides lutzii* (*Pb*01)	“*Pb*01-like”	[31]
*Paracoccidioides brasiliensis* (*Pb*18)	S1	[26]
*Paracoccidioides brasiliensis* (*Pb*02)	PS2	[26]
*Paracoccidioides brasiliensis* (*Pb*Epm83)	PS3	[27]

*A. nidulans* Δ*sidA* strain [16] was grown at 37°C in *Aspergillus* minimal medium (AMM), as described [41], containing 1% glucose as carbon source, 20 mM glutamine as the nitrogen source, 10 µM FeSO_4_, 20 µg l^−1^ biotin and 10 µM triacetylfusarinine C (TAFC).

### RNA isolation and quantitative real time PCR (qRT-PCR)

*Pb*01yeast cells were incubated in MMcM supplemented with 50 µM BPS or in MMcM containing 3.5 µM ammonium ferrous sulfate. Cells were collected after 24 h and total RNA was isolated using trizol (TRI Reagent, Sigma-Aldrich, St. Louis, MO) and mechanical cell rupture (Mini-Beadbeater - Biospec Products Inc., Bartlesville, OK). RNAs were reverse-transcribed using SuperScript III First-Strand Synthesis SuperMix (Invitrogen, Life Technologies) and cDNAs were submitted to qRT-PCR in the StepOnePlus real-time PCR system (Applied Biosystems Inc.). SYBR green PCR master mix (Applied Biosystems, Foster City, CA) was used in the reaction mixture and the PCR thermal cycling was 40 cycles of 95°C for 15 s and 60°C for 1 min. The sequences of forward and reverse oligonucleotides used are listed in **Table S1**. One primer in each pair spanned an intron, preventing amplification from genomic DNA. The qRT-PCR reaction was performed in triplicate for each cDNA sample and a melting curve analysis was accomplished to confirm a single PCR product. The data were normalized with the transcript for α-tubulin (GenBank accession number XM_002796593) amplified in each set of qRT-PCR experiments. A non-template control was included. A relative standard curve was generated by pooling an aliquot from each cDNA sample which was serially diluted 1∶5 to 1∶125. Relative expression levels of transcripts of interest were calculated using the standard curve method for relative quantification [42]. Student's *t*-test was applied in the statistical analyses and *P* values of 0.05 or less were considered statistically significant.

### Upstream sequence analysis

Upstream regions of siderophore biosynthesis and *mirB* genes of *Pb*01 were inspected for the presence of conserved sequences related to iron regulated transcription of siderophore genes. For all genes (*sidD*, *sidF*, *sidA*, *sidI* and *mirB*) the upstream region comprehends the entire intergenic region from the 5′open reading frame (**Figure S2**).

### *In silico* analysis of putative *Paracoccidioides sidH* and *sidI* orthologs

The amino acid sequences of putative *Paracoccidioides sidH* and *sidI* orthologs were obtained at the Dimorphic Fungal Database of the Broad Institute site (http://www.broadinstitute.org/annotation/genome/dimorph_collab//MultiHome.html) based on homology search. The sequences have been submitted to GenBank with the following accession numbers SidH: *Pb*01 (XP_002791730), *Pb*18 (EEH45393) and *Pb*03 (EEH20785); SidI: *Pb*01 (XP_002796673), *Pb*18 (EEH43810) and *Pb*03 (EEH21513). The amino acid sequences of *Paracoccidioides* and *A. fumigatus* (SidH XP_748661; SidI XP_753087) orthologs were aligned using CLUSTALX2 [43]. The peroxisomal targeting signal 1 (PTS1) scores of proteins were obtained using the PTS1-predictor program http://mendel.imp.ac.at/mendeljsp/sat/pts1/PTS1predictor.jsp [44]. Positive scores indicate high probability of peroxisomal targeting. Peroxisomal targeting signal 2 (PTS2) motifs were identified using the PTS2 finder http://www.peroxisomedb.org/diy_PTS2.html.

### Chrome azurol S (CAS) assays

Siderophore production by *P. lutzii* and *P. brasiliensis* was qualitatively analyzed with an overlay-CAS (O-CAS) as described [45]. *Pb*01, *Pb*18, *Pb*02 and *Pb*Epm83 yeasts were grown for 13 days at 36°C on MMcM agar plates, without iron addition. For iron sufficiency (control), ammonium ferrous sulfate was used in a final concentration of 30 µM. CAS medium was prepared according to [46] with minor modifications. Briefly, 100 ml of O-CAS was prepared with 6.05 mg CAS dissolved in 5 ml water and mixed with 83.2 µl of ferric chloride solution (30 mM FeCl_3_. 6 H_2_O in HCl 10 mM). Under stirring this solution was slowly added to 7.29 mg hexadecyltrimetyl ammonium bromide (HDTMA) dissolved in 4 ml water. The resultant dark blue liquid was autoclaved at 121°C for 15 min. A mixture of 3.024 g piperazine-1,4-bis(2-ethanesulfonic acid) (PIPES) dissolved in 75 ml water (pH 6.8) was also autoclaved with agarose (0.9%, w/v) as the gelling agent. After cooling to 50°C, both PIPES and dye solutions were mixed with enough care to avoid foaming. After that, 15 ml of O-CAS were applied over the plates in order to detect secreted siderophores. The ternary complex chrome azurol S/Fe^3+^/HDTMA serves as an indicator. When a strong chelator, such as siderophores, removes the iron from the dye its color turns from blue to orange, which indicates the presence of hydroxamates according to Perez-Miranda and co-workers [45].

The percent siderophore activity in *Pb*01 and *Pb*18 supernatants was determined as described [47]. Yeast cells were cultured at 36°C in MMcM liquid medium with no iron addition and MMcM containing 30 µM ammonium ferrous sulfate. Supernatants were collected after 6, 10 and 15 days of incubation. After sterile filtration with 0.22 µM pore filter, 500 µl of supernatants as well as a reference prepared with non-inoculated MMcM were added to 500 µl of CAS liquid medium also prepared according to Schwyn and Neilands [46]. Briefly, 6 ml of 10 mM HDTMA solution was placed in 100 ml volumetric flask. A mixture of 1.5 ml of ferric chloride solution (3 mM FeCl_3_. 6 H_2_O in HCl 10 mM) and 2 mM aqueous CAS solution was slowly added to the HDTMA flask under stirring. An aqueous solution containing 4.307 g PIPES (pH 5.6) was added to the volumetric flask which was then filled with water to afford 100 ml of CAS assay solution. The mixture of CAS-supernatants (s) and CAS-reference (r) was incubated at room temperature and absorbance at 630 nm was measured after 1 h (Ultraspec 2000 UV/Visible Spectrophotometer, Pharmacia Biotech). The percent siderophore activity was calculated by subtracting the sample absorbance values from the reference according to the formula [(A_r_–A_s_/A_r_)] ×100.

Glassware was acid treated to remove residual traces of iron [48]. All the reagents used for CAS medium preparation were purchased from Sigma-Aldrich, St. Louis, MO.

### Ferric perchlorate assay

The presence of hydroxamates in the *Pb*01 and *Pb*18 supernatants was checked with the colorimetric ferric perchlorate assay [49]. Yeast cells were cultivated for 10 days at 36°C in MMcM liquid medium with no iron addition and MMcM containing 30 µM ammonium ferrous sulfate. After sterile filtration with 0.22 µM pore filter, an aliquot of each supernatant was lyophilized. Samples were then dissolved and concentrated to one tenth of the original volume with MilliQ-water. The volume of 1.25 ml of 5 mM Fe(ClO_4_)_3_ in 0.1 M HClO_4_ solution was added to 250 µl of concentrated supernatants, as well as to a reference prepared with sterile MMcM, and allowed to incubate at room temperature for approximately five minutes. The formation of an orange-red color demonstrates the presence of hydroxamates.

### Isolation and identification of siderophores

For isolation and characterization of *Pb*01 and *Pb*18 secreted siderophores, yeasts cells were cultivated for 4, 10 and 18 days in MMcM medium with no iron addition. Culture supernatants were filtered (0.22 µM) and lyophilized. Samples were then dissolved and concentrated to one tenth of the original volume with MilliQ-water. 250 µl of 100 mM FeSO_4_ was added to the samples in order to convert desferri-siderophores in ferri-ones. An aliquot of 2.5 ml was applied to an Amberlite XAD-16 column (Rohm and Haas, Philadelphia, PA, USA). Siderophore-iron complexes were eluted with 2 ml of methanol and collected. Methanol was discarded by speed vacuum centrifugation overnight. The dried pellet was solubilized in 100 µl of water and 10 µl were applied to reversed phase HPLC (RP-HPLC). Samples were separated using a Nucleosil 100-5 C_18_ column (250 mm×4 mm I.D.; 5 µm particle pore size; Macherey-Nagel, Düren, Germany). Chromatography was performed within 40 min at a constant flow of 0.5 ml min^-1^ with a two-step acetonitrile gradient starting at solvent A - solvent B (94∶6) (solvent A: water containing 0.1% TFA; solvent B: 85% acetonitrile and 0.1% TFA). The concentration of solvent B was increased linearly from 6% to 15% during 10 min, from 15% to 60% during 25 min and held at 60% for 5 min. Fractions obtained in this way were collected, lyophilized and stored at −20°C.

Determination of the molecular mass of the samples obtained by RP-HPLC was carried out using a LTQ Velos ion trap mass spectrometer (Thermo Fisher Scientific) equipped with an electrospray source (ESI-MS, Electrospray Ionization Mass Spectrometry). Samples were dissolved in 50% aqueous methanol containing 0.1% formic acid, and infused directly into the ion source using the syringe pump. The electrospray voltage was set at 4.0 kV and the heated capillary was held at 270°C.

For analysis of cellular siderophores, equal number of *Pb*01 and *Pb*18 yeast cells was cultivated for 8 days in MMcM medium with no iron addition. Cells were harvested by centrifugation and washed five times with PBS 1X in order to get rid of extracellular siderophores. Subsequently, cellular extracts were prepared by grinding yeast cells into a fine powder using a mortar and pestle under liquid nitrogen. The powder was resuspended in water (1 ml sterile water/4 ml culture) and the suspension was centrifuged. Cellular debris were discarded, the supernatants were filtered (0.22 µM) and lyophilized. Samples were dissolved and concentrated to one tenth of the original volume with MilliQ-water and analyzed as described for the extracellular siderophores.

### Macrophage infection experiments

Murine macrophage cell line J774 A.1 (BCRJ Cell Bank, Rio de Janeiro, accession number 0121) maintained in RPMI medium (RPMI 1640, Vitrocell, Brazil) supplemented with non-essential amino acids (M7145 Sigma-Aldrich, St. Louis, MO), 10% (v/v) fetal bovine serum (FBS), at 37°C in 5% CO_2_, were used in the assays. 1×10^6^ macrophages were seeded into each well of a 24-well tissue culture plate and 100 U ml^−1^ of murine gamma interferon (IFN-γ; PeproTech, Rocky Hill, New Jersey, USA) was added for 24 h at 37°C in 5% CO_2_ for macrophage activation as described [50].

*Paracoccidioides* yeast cells exposed to siderophores were co-cultivated with activated macrophages and the number of viable fungal cells after phagocytosis was assessed by colony forming unit (CFU) counts. Briefly, *Pb*01 and *Pb*18 were incubated in MMcM with no iron addition and containing 50 µM of BPS for 24 h under rotation. An equal number of fungal cells was next exposed to 10 µM of each ammonium ferrous sulfate, DA and FO for 3 h. Additionally, yeast cells were also incubated in MMcM with no iron addition and containing 50 µM BPS. 2×10^6^ *Paracoccidioides* viable yeasts cells were then added to the wells containing 1×10^6^ macrophages (yeast-to-macrophage ratio 2∶1). The cells were co-cultivated for 24 h at 37°C in 5% CO_2_ to allow fungal internalization. Each well was washed twice with 1 ml PBS 1X in order to get rid of non-internalized yeasts. Infected macrophages were lysed with water and dilutions of the lysates containing the phagocytized yeasts were plated on BHI medium supplemented with 4% (v/v) sheep blood and 4% glucose. After incubation at 36°C for 9 days, the number of CFU was determined to check the ability of yeast cells exposed to siderophores to survive in macrophages. CFU were expressed as the mean value ± the standard error from triplicates. Student's *t*-test was applied in the statistical analyses and *P* values of 0.05 or less were considered statistically significant.

For gene expression analysis, *Pb*01 was grown in BHI and, after three washes with PBS1X, 2×10^6^ viable yeasts cells were incubated with 1×10^6^ activated macrophages in presence of 50 µM BPS (added to the RPMI immediately before addition of yeast cells). Cells were co-cultivated for 24 h at 37°C in 5% CO_2_ to allow fungal internalization. Each well was washed twice with 1 ml PBS 1X in order to get rid of non-internalized yeasts. Trizol was added to each well and total RNA of internalized yeasts was isolated. RNAs from uninfected macrophages and from *Pb*01 yeast cells cultured in RPMI 1640 medium, also in presence of BPS, were obtained as control. After reverse transcription, cDNAs were submitted to qRT-PCR, as described above.

### Cross-feeding experiments

*Pb*01 and *Pb*18 yeasts cells were incubated in MMcM with no iron addition and containing 50 µM BPS. After 24 h, 1×10^7^ yeasts cells were spotted on MMcM supplemented with 200 µM BPS and incubated at 36°C for 7 days. Next, 1×10^7^ *A. nidulans ΔsidA* spores were point-inoculated 2 cm distant from the borders of *Paracoccidioides* colonies and plates were incubated for 48 h. As control, *A. nidulans ΔsidA* spores were also spotted on MMcM 200 µM BPS plates in the absence of *Paracoccidioides* yeasts.

## Results

### Genomic organization and identification of putative regulatory sites of *Paracoccidioides* siderophore genes

**Figure 1:**
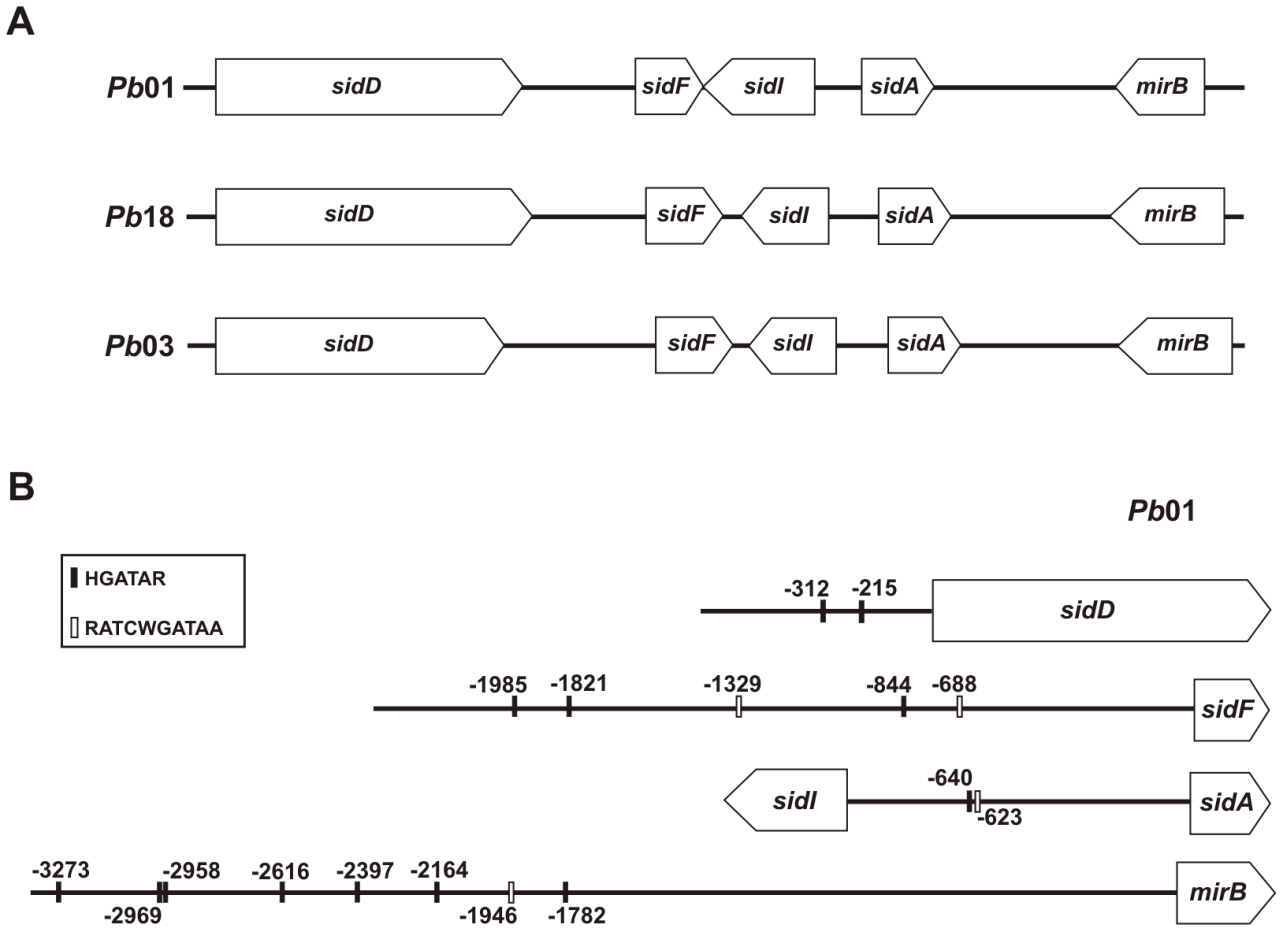
Genomic organization and regulatory sites in upstream regions of siderophore genes. A: Representation of siderophore biosynthesis and uptake genes localization in *Pb*01, *Pb*18 and *Pb*03 genomes. B: Schematic figure showing the position and sequence of putative regulatory sites in upstream regions of siderophore genes in *Pb*01. A black line represents nucleotides (nt) located 5′ to the open reading frame of each gene: *sidD* 679 nt, *sidF* 2409 nt, *mirB* 3376 nt. The intergenic region between *sidI* and *sidA* includes 1005 nt. Numbers represent the number of nucleotides upstream the start codon of each gene where the regulatory sequence was found (vertical line). In case of divergent genes, the nucleotide position is relative to *sidA*. H: A/T/C; R: A/G; W: A/T. Accession numbers are available in Table S2.

Genes involved in the siderophore biosynthesis pathway tend to be genomically clustered [51] and a similar pattern of organization was found in *Paracoccidioides*. Four out of the six iron regulated biosynthetic genes (including *sidA*) are located next to each other in a region of approximately 22 kb of *Pb*01 genome (**Figure 1A**), which interestingly also includes the putative siderophore transporter-encoding gene *mirB*. The gene cluster organization of these iron regulated genes was also found in *Pb*18 and *Pb*03 genomes.

The 5′ upstream regions of the siderophore biosynthetic cluster (**Figure S2**) were examined in order to identify conserved sequences. In many pathogenic and non-pathogenic fungi the GATA sequences localized in the promoter regions of siderophore metabolism genes are recognized by GATA-type transcription factors that promote repression of siderophore synthesis under iron sufficiency [52], [53], [54], [55]. As depicted in **Figure 1B**, the upstream regions of all iron regulated genes presented at least one HGATAR motif [56]. An extended version of the HGATAR, the 5′-(G/A)ATC(T/A)GATAA-3′ consensus sequence, formerly identified in the 5′regions of the siderophore biosynthetic cluster in *Histoplasma capsulatum* [21] and *A. fumigatus* [53], was also found in the upstream regions of the siderophore gene cluster in *Paracoccidioides*, excepting *sidD*.

### Transcriptional analysis of siderophore orthologs genes

**Figure 2:**
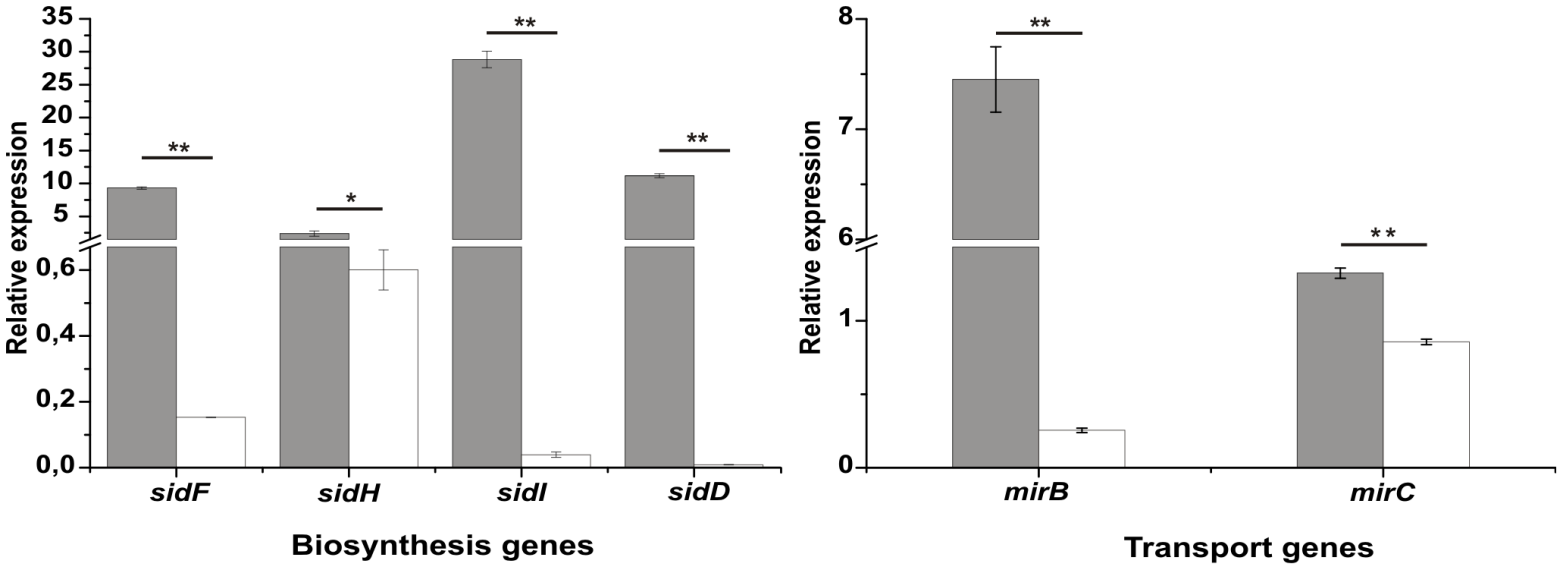
Low iron availability induces expression of putative siderophore biosynthesis and uptake genes. Quantitative RT-PCR was performed with transcripts of *Pb*01 yeast cells under iron-limited conditions (50 µM BPS; gray bars) and incubated with 3.5 µM ammonium ferrous sulfate (white bars). Expression values of siderophore biosynthesis (*sidF*, *sidH*, *sidI*, *sidD*) and transport genes (*mirB*, *mirC*) were calculated using α-tubulin as endogenous control. Data are expressed as mean + standard deviation from triplicates. Statistically significant difference was determined by Student's *t*-test (*p<0.05/ ** p<0.001).

Analysis performed by Parente et al. [33] showed an induction of *Pb*01 genes putatively related to siderophore biosyntheis (*sidA*) and uptake (*sit1*) under iron limiting conditions, indicating that *Paracoccidioides* may produce and capture siderophores to overcome iron starvation. To verify that the other orthologs genes related to siderophore biosynthesis and uptake are also transcriptionally regulated by iron availability, quantitative RT-PCRs (qRT-PCRs) were carried out. Most of the analyzed transcripts, *sidF*, *sidH*, *sidI*, *sidD*, *mirB* and *mirC*, were induced under iron limiting conditions (50 µM BPS) after 24 h of incubation, as depicted in **Figure 2**. The expression of *sidC* was not significantly affected under iron limiting conditions (data not shown). This is probably related to its putative function in the synthesis of intracellular siderophores, as described in *A. fumigatus* [17]. The *sidI* and *sidH* genes encode an acyl-CoA ligase and an enoyl-CoA hydratase, respectively. Both enzymes link ergosterol and siderophore biosynthesis in *A. fumigatus* [20] and are found in most fungi that produce siderophores. BlastP (http://blast.ncbi.nlm.nih.gov/Blast.cgi) searches retrieved *Pb*01, *Pb*18 and *Pb*03 sequences presenting, respectively, 68, 65 and 65% identity with *A. fumigatus* SidI (**Figure S3**). The SidH orthologs from *A. fumigatus* and *Pb*01, *Pb*18, *Pb*03 share, respectively 52, 47 and 47% identity at the amino acid sequence level (**Figure S4**). As in other *Ascomycetes* [51], SidI and SidH from *Paracoccidioides* also carry putative peroxisomal targeting sinal (PTS) motifs. PTS2 was found in all *Paracoccidioides* SidI and PTS1 is present in all *Paracoccidioides* SidH (**Figure S3 and S4**). The latter indicates that siderophore biosynthesis is partially localized in peroxisomes as previously shown in *A. nidulans* and *A. fumigatus* [51]. Taken together, the presence of orthologs involved in siderophore biosynthesis and uptake and their induction during iron-deficient conditions suggest that *Paracoccidioides* is a siderophore producer.

### Detection of secreted siderophores in *Paracoccidioides* cultures

**Figure 3:**
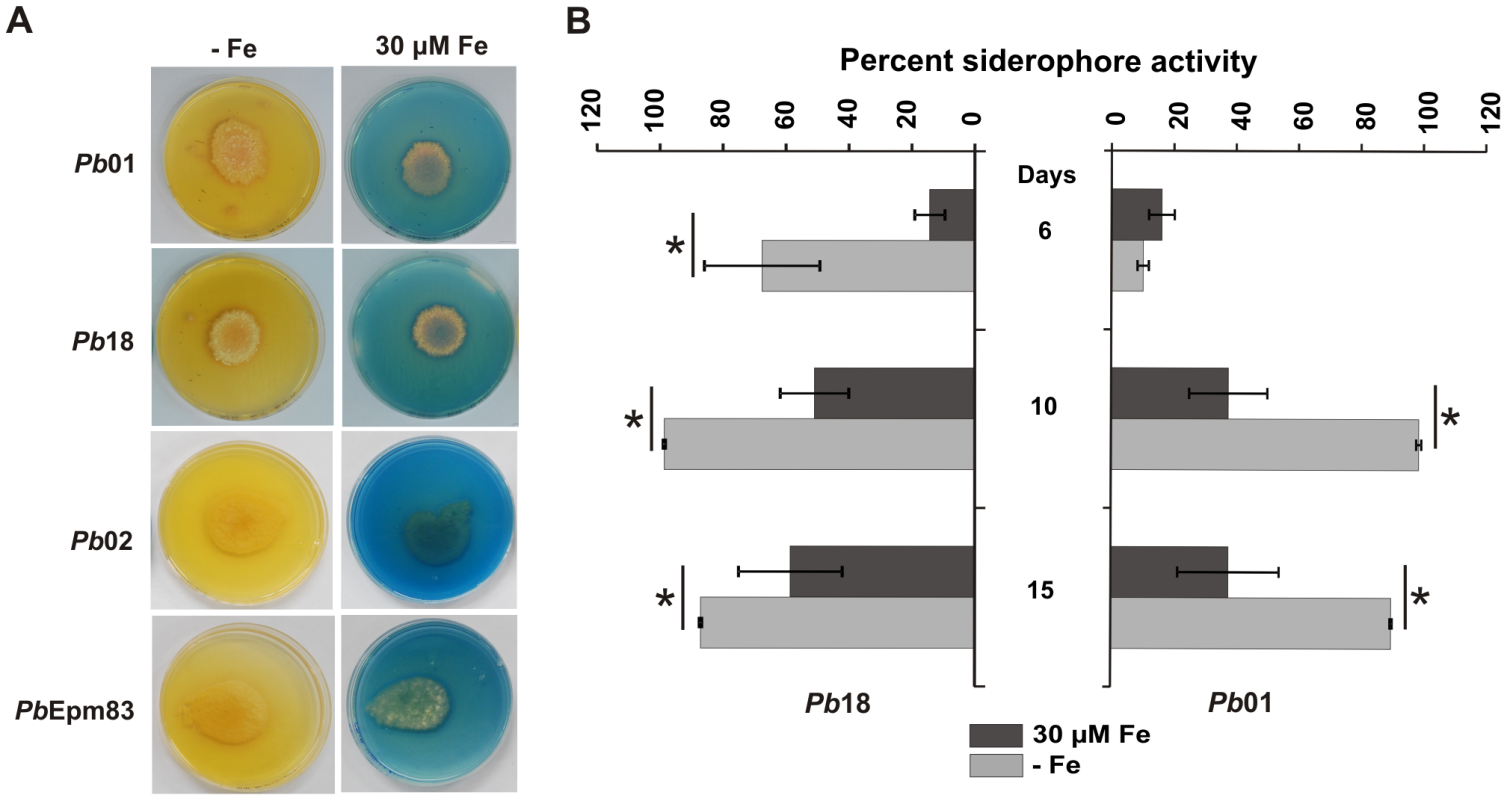
Iron limiting conditions induce biosynthesis and secretion of siderophores. A: Detection of siderophores in MMcM agar plates. Under iron sufficiency (30 µM Fe), the production of these chelators was repressed in solid medium. B: Percent siderophore activity was determined in *Pb*01and *Pb*18 supernatants. Values are expressed as mean + standard deviation of three experiments. *statistically significant difference determined by Student's *t*-test (p<0.05).

Since orthologs genes for siderophore production were induced under iron limiting conditions, *Paracoccidioides* cultures were examined for the presence of these molecules by using the CAS detection medium [46]. *Pb*01, *Pb*18, *Pb*02 and *Pb*Epm83 yeasts were incubated at 36°C on MMcM agar plates with no iron addition or with 30 µM ammonium ferrous sulfate. After 13 days, an overlay-CAS (O-CAS) was added to the plates and a change in color from blue to orange was observed after a few hours in the iron depleted plates (**Figure 3A**), which indicates the secretion of siderophores [45]. The presence of hydroxamate-type siderophores was confirmed with the ferric perchlorate assay, as shown in **Figure S5**.

The percent siderophore activity was then determined in *Pb*01 and *Pb*18 supernatants, by mixing it with CAS, after 6, 10 and 15 days of incubation in MMcM. As shown in **Figure 3B**, siderophores were produced in higher amounts in 10 and 15 days of growth under iron limiting conditions. An increase in biomass was perceived during incubation in both solid and liquid iron poor media (data not show), demonstrating that *Paracoccidioides* possesses a functional high affinity iron uptake mechanism.

### Identification and characterization of *Paracoccidioides* siderophores

**Figure 4:**
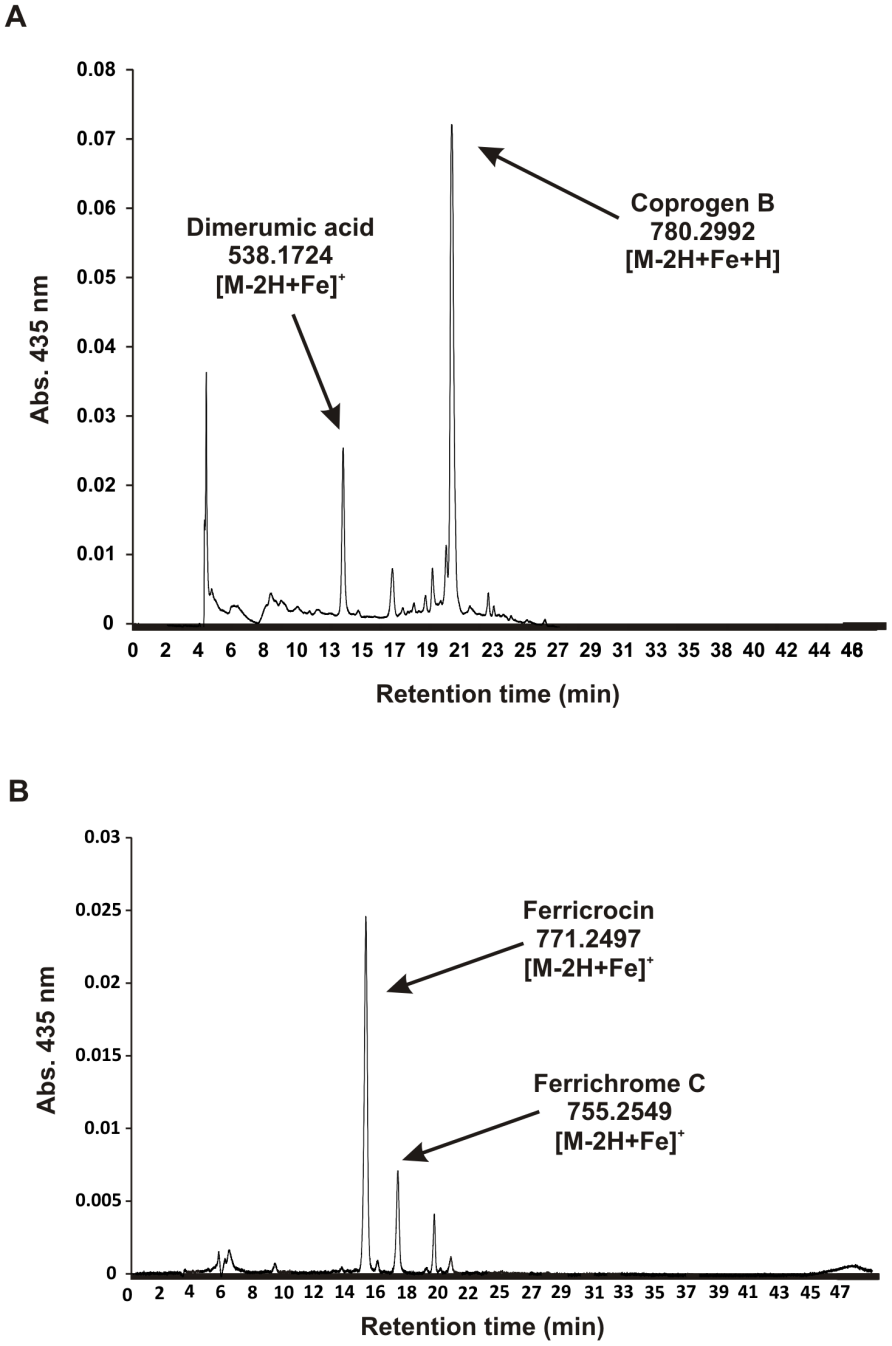
Identification of extra- and intracellular siderophores produced by *Paracoccidioides*. A: Reversed-phase HPLC of *Pb*01culture supernatants after 4 days of incubation under iron limiting conditions. Coprogen B and dimerumic acid were identified as extracellular siderophores. B: RP-HPLC of *Pb*18 cell extracts obtained after cultivation for 8 days in iron depleted medium. The intracellular siderophores identified were ferricrocin and ferrichrome C. Molecular masses of iron bound compounds are indicated.

In order to identify the siderophores detected by the CAS solution, *Pb*01 and *Pb*18 yeasts were grown under iron limiting conditions and supernatants were analyzed by RP-HPLC and mass spectrometry. Following addition of FeSO_4_, reversed-phased HPLC showed that compounds exhibiting absorption at 435 nm were secreted by both *Pb*01 and *Pb*18 under iron depleted conditions. **Figure 4A** shows HPLC analysis of *Pb*01 culture supernatants after 4 days of cultivation. High-resolution mass spectrometry of RP-HPLC peaks displaying absorption at 435 nm, which is typically for iron-saturated siderophores, yielded the molecular masses m/z (M-2H+Fe)^+^ = 538.1724 matching C_22_H_36_N_4_O_8_Fe (dimerumic acid, calculated molecular mass 538.1717) and (M-2H+Fe)^+^ = 780.2992 matching C_33_H_54_N_6_O_12_Fe (coprogen B, calculated molecular mass 780.2979). Notably, dimerumic acid is most likely a breakdown product of coprogen B. In agreement, tandem mass spectrometry (MS/MS) of coprogen B generated dimerumic acid (**Figure S6A**). Further analysis of *Pb*01 and *Pb*18 supernatants from 10 days of growth under iron limiting conditions reinforced that dimerumic acid is a breakdown product of coprogen B, since the amount of coprogen B decreased while dimerumic acid increased compared to 4 days supernatants (**Figure S6B**). Also, after 18 days, only dimerumic acid was found (data not shown).

Some ascomycetes are known to produce intracellular siderophores for iron storage [57], [58]. To further investigate the production of these molecules in the *Paracoccidioides* genus, cell extracts of *Pb*01 and *Pb*18 were prepared as described in *Materials and Methods* and submitted to reversed-phased HPLC. RP-HPLC peaks displaying absorption at 435 nm were seen in both *Pb*01 and *Pb*18 cell extracts. **Figure 4B** depicts HPLC analysis of *Pb*18 exemplary. The high resolution mass spectrometry analysis unambiguously identified the major intracellular siderophores as ferricrocin (C_28_H_47_N_9_O_13_Fe) and ferrichrome C (C_28_H_47_N_9_O_12_Fe). As seen in **Figure S7**, both siderophores were detected with four different ionizing adducts: H^+^ (m/z = 771.2497 for ferricrocin and m/z = 755.2549 for ferrichrome C), NH4^+^ (m/z = 788.2768 for ferricrocin and m/z = 772.2818 for ferrichrome C), Na^+^ (m/z = 793.2320 for ferricrocin and m/z = 777.2371 for ferrichrome C) and K^+^ (m/z = 809.2060 for ferricrocin and m/z = 793.2111 for ferrichrome C). Additional peaks arise from ^13^C and ^54^Fe isotopic variants. MS/MS fragmentation analysis of the here identified ferricrocin from *Paracoccidioides* resembles that of *A. fumigatus* ferricrocin (data not shown). Moreover, comparison of MS/MS fragmentation analysis of the presumable ferricrocin and ferrichrome C from *Paracoccidioides* also support their identity (**Figure S8**).

### Growth of *Paracoccidioides* in presence of siderophores as iron sources

**Figure 5:**
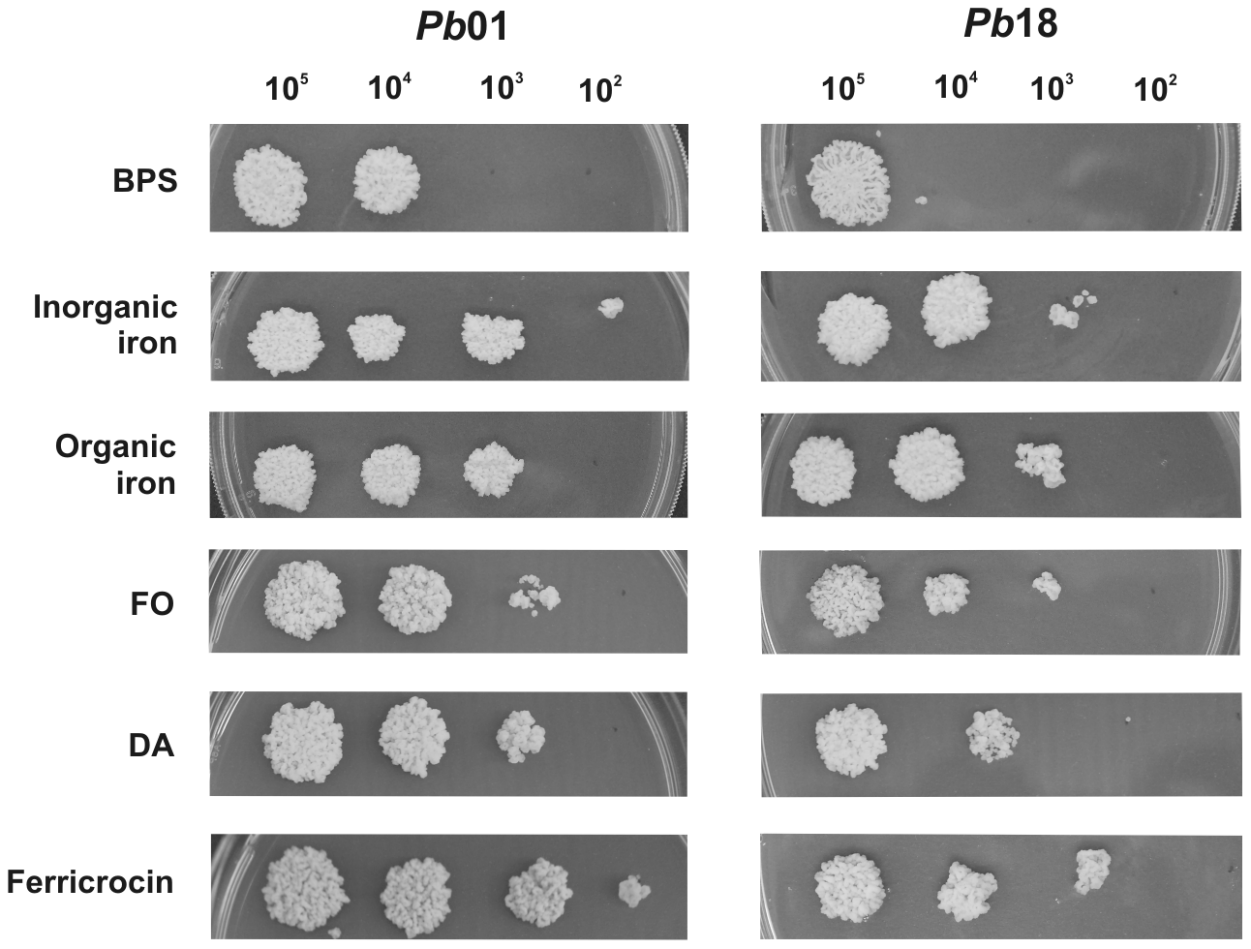
Effect of siderophores on *Paracoccidioides* growth. After growth on MMcM + BPS for 24 h, *Pb*01 and *Pb*18 yeast cells were washed and serially diluted. 10^5^ to 10^2^ cells were spotted on MMcM agar plates containing 50 µM BPS only, 50 µM BPS plus 10 µM of siderophores and 10 µM of inorganic and organic iron (without BPS). Inorganic iron: ammonium ferrous sulfate; organic iron: ammonium ferric citrate; DA: dimerumic acid; FO: ferrioxamine.

The presence of putative siderophore transporters in the *Paracoccidioides* genomes and its induction under iron limiting conditions suggest that the fungus besides producing, is able to utilize siderophores as well. To test this hypothesis, serial dilutions of *Pb*01 and *Pb*18 yeasts cells were spotted on MMcM agar plates containing BPS and supplemented or not (iron limiting conditions) with hydroxamate siderophores, as well as with organic and inorganic iron compounds (not supplemented with BPS). Although minor differences were seen in the growth profile, both strains grew in presence of siderophores as the only iron sources, as show in **Figure 5**. This suggests that *Paracoccidioides* may be able to uptake siderophore-iron complexes from the extracellular environment and, subsequently, utilize the iron for metabolism and growth. Residual growth in presence of BPS is not surprising, since this ferrous iron-specific chelator does not affect siderophore-iron utilization.

### Infection of murine macrophages cell line with *Paracoccidioides* yeasts cells exposed to siderophores

**Figure 6:**
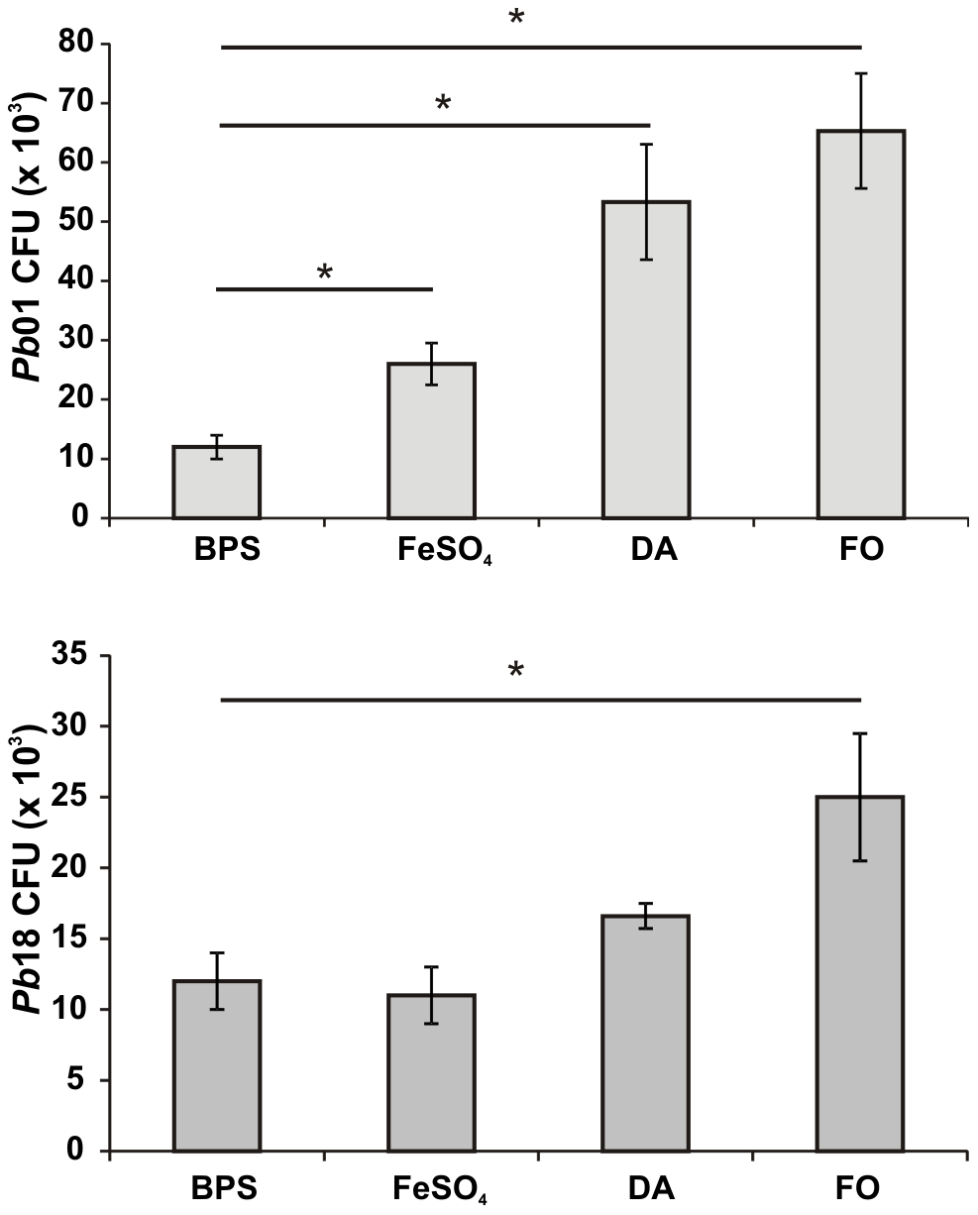
Exposure to siderophores increases *Paracoccidioides* ability to survive macrophage killing. Prior exposure to 10 µM FO and DA enhanced *Pb*01 and *Pb*18 resistance to macrophage killing. CFU counts are expressed as mean + standard error from triplicates, representative of two independent experiments. *statistically significant difference determined by Student's *t*-test (p<0.05).

Lung resident macrophages are the first defense cells which interact with *Paracoccidioides* following host invasion. It was demonstrated that fungal survival within human monocytes is iron dependent since the inhibitory effect of the iron chelator deferoxamine is reversed by holotransferrin [59]. IFN-γ and other cytokines modulate cellular iron metabolism to strengthen host iron withholding defenses, culminating in reduced iron availability to pathogens inside macrophages [60]. As iron is critical for *Paracoccidioides* yeasts survival in monocytes, the susceptibility of yeast cells exposed to siderophores to killing by IFN-γ activated macrophages was evaluated. Following growth on iron limiting conditions (50 µM BPS), *Pb*01 and *Pb*18 yeasts cells were incubated in the presence of DA, FO and ammonium ferrous sulfate, and in the absence of any iron source (addition of 50 µM BPS), prior to co-cultivation with macrophages. As shown by CFU counting, *Pb*01 and *Pb*18 yeasts cells survival in infected macrophages increased following exposure to DA and FO, when compared to ammonium ferrous sulfate and incubation in presence of BPS (**Figure 6**). Thus, the increased ability to survive to macrophage killing was probably a result of siderophore-iron utilization.

### Transcriptional analysis of putative siderophore biosynthesis *sidA* gene during infection of murine macrophages cell line

**Figure 7:**
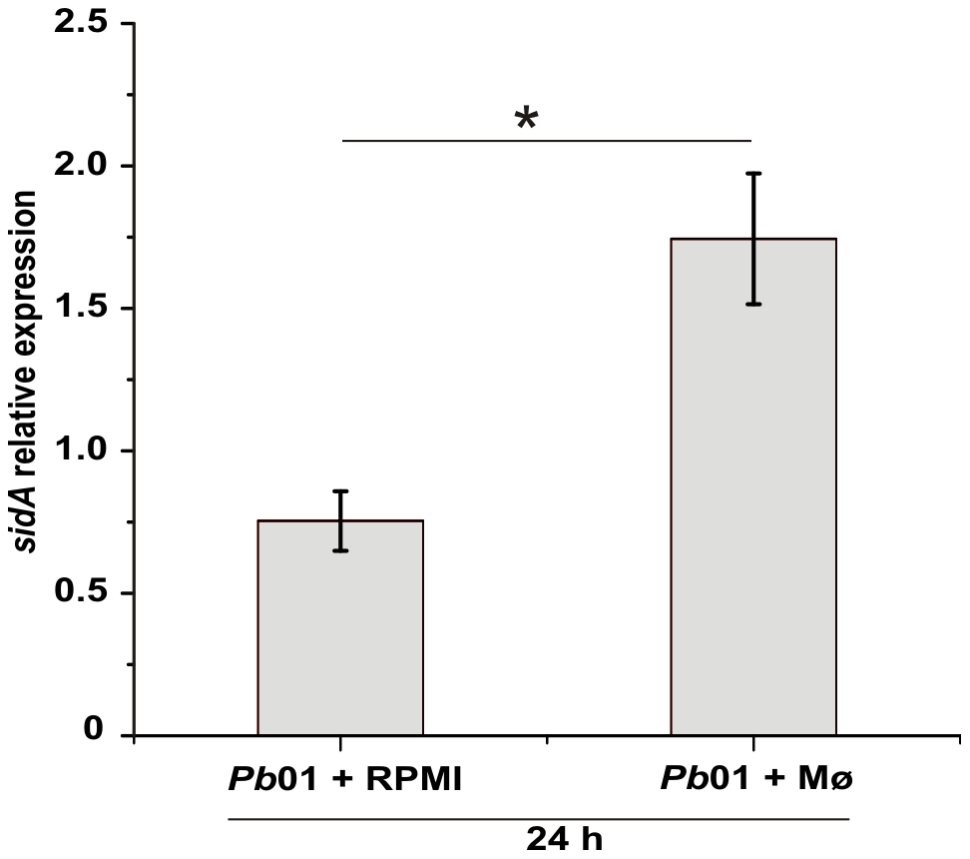
*Paracoccidioides sidA* expression is induced during murine macrophage infection. Quantitative RT-PCR was performed with transcripts of *Pb*01 yeast cells phagocytosed by murine macrophages after 24 h of co-incubation. As control, yeast cells were incubated for 24 h in RPMI medium. 50 µM BPS was added to the RPMI medium in both conditions. Expression values were calculated using α-tubulin as endogenous control. Data are expressed as mean + standard deviation from triplicates. *statistically significant difference determined by Student's *t*-test (p<0.001).

Taking into account the putative role of *Paracoccidioides sidA* gene in siderophore production and its induction under iron limiting conditions *in vitro* [33], the expression of this gene was examined in *Pb*01 yeasts cells after co-cultivation with murine macrophages. Following growth to exponential phase, viable yeasts were co-cultivated with IFN-γ activated macrophages and, after 24 h, RNAs from phagocytosed yeasts were obtained. As shown **Figure 7**, the abundance of *sidA* transcripts increased 2.3 times during co-cultivation with macrophages when compared to the non-infectious condition. This indicates that *sidA* may play a relevant role during fungus-macrophage interaction.

### Cross-feeding between *Paracoccidioides* and *A. nidulans* Δ*sidA* mutant

**Figure 8:**
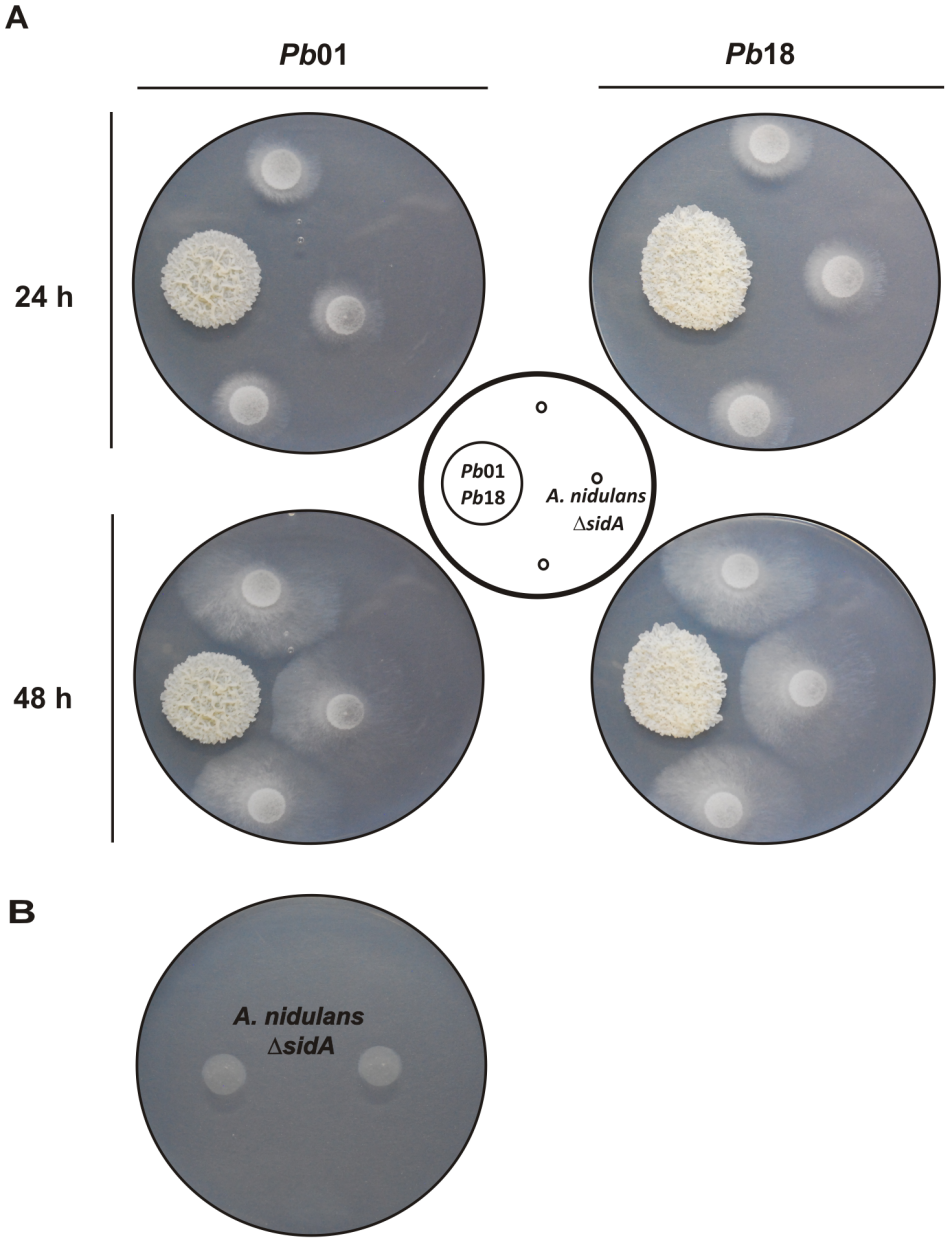
*Paracoccidioides* siderophores promote growth of *A. nidulans ΔsidA* mutant. A: *Aspergillus* hyphae grew on plates containing *Pb*01 and *Pb*18 yeasts cells grown under iron limiting conditions. B: No growth was observed in absence of *Paracoccidioides*.

Siderophore utilization is not restricted to the producing microorganisms. Several bacteria and fungi can take up and utilize iron bound to siderophores produced by other microbial species (xenosiderophores). *In vitro* growth and CFU recovery from co-cultivation with murine macrophages suggest that *Paracoccidioides* may utilize the xenosiderophore ferrioxamine and DA as iron sources. To check if *Paraccoccidioides* siderophores could be utilized as iron sources by other fungal species, growth of *A. nidulans ΔsidA* strain, which is unable to produce these molecules, was tested. Spores of *A. nidulans ΔsidA* mutant were point-inoculated in vicinity to 7 days-old colonies of *Pb*01 and *Pb*18 on plates (MMcM+200 µM BPS). As shown in **Figure 8A**, in 24 h *Aspergillus* hyphae started growing toward *Paracoccidioides* colonies and the radial growth was sustained for the next 24 h. This suggests that siderophores secreted by *Paracoccidioides* promoted the growth of *ΔsidA* mutant, since no growth was observed in absence of *Paracoccidioides* (**Figure 8B**).

## Discussion

As for other organisms, iron is also essential for growth of *Paracoccidioides* [32]. Since this fungus is the causative agent of a systemic mycosis, it faces host iron-withholding and must be able to overcome this condition in order to establish the infection. The knowledge of the strategies used by pathogenic microorganisms to acquire iron is extremely important to understand the host-pathogen interaction and, consequently, for the treatment of the disease. Nevertheless, information about the mechanisms developed by *Paracoccidioides* for iron uptake is still scarce.

In an effort to start deciphering the molecular mechanisms employed by this fungus for iron acquisition, we firstly screened its genomic sequence for genes putatively involved in iron homeostasis. Based on homology analysis and precedent from other fungi, orthologs of genes related to high affinity iron uptake systems, including those for siderophore biosynthesis and uptake, were found [39]. Here we show that these genes putatively involved in siderophore production and utilization were transcriptionally induced under iron limiting conditions, which is in agreement with their possible role in a high affinity uptake system.

Genes encoding proteins, which are involved in a common metabolic pathway, tend to be clustered in the genome. Indeed, most *Paracoccidioides* siderophore biosynthetic genes were co-localized in a genome region that also included the transport gene *mirB*. We have previously shown that MirB amino acid sequence presents a major facilitator superfamily (MFS) domain (MFS1) [39]. Interestingly, a MFS transporter (*MFS2*) was also found within an iron regulated siderophore biosynthetic gene cluster in the closely related pathogen *H. capsulatum* [21]. Taking into account the organization in cluster and the co-regulation of these genes in response to iron levels, their upstream regions were inspected for the presence of conserved sequences. The HGATAR motif and the RATCWGATAA consensus sequence were found. As the GATA sequences are target of transcription factors that regulate siderophore biosynthesis, we hypothesize that *Paracoccidioides* siderophore genes could also be target of regulation by these factors.

Taken together, the presence and putative regulatory elements of siderophore biosynthetic genes strongly suggested that *Paracoccidioides* could be a siderophore producer. The presence of these molecules was firstly detected in iron deprived *Paracoccidioides* agar plates with an overlay of CAS solution, which suggested the presence of hydroxamates, siderophores typically produced by fungi [14]. Even when iron was omitted from the culture medium, the production and secretion of desferri-hydroxamates, which extracellularly chelated traces of iron, and the subsequent uptake of the iron-siderophore complex allowed fungal growth. Siderophore production by *Paracoccidioides* was more affected by the iron addition in solid than in liquid medium. Iron at 30 µM completely abrogated siderophore production in agar plates, but the same concentration did not impair siderophore secretion in culture supernatants. Growth rate on liquid medium is higher than in solid. As the utilization of iron increases according to the incubation time, due to the consumption by the growing number of cells, the availability of this nutrient in the culture decreases and induces the production and secretion of siderophores, even if iron was added initially. The same differences in siderophore production were observed in *Aspergillus* species [47].

Fungal hydroxamate siderophores can be classified into four structural families: coprogens, ferrichromes, fusarinines and rhodotorulic acid. Reversed-phase HPLC and mass spectrometry analysis allowed the confirmation of hydroxamate production by *Paracoccidioides*. Coprogen B and dimerumic acid were identified as extracellular siderophores while ferrichrome C and ferricrocin as the intracellular ones. Coprogen-type siderophores contains anhydromevalonyl residues linked to the hydroxylated ornithine by the action of the transacylase SidF. This acyl group derives from mevalonate by CoA ligation and dehydration catalyzed, respectively, by SidI and SidH [20]. Accordingly, the presence of orthologs to *sidI*, *sidH* and *sidF* in *Paracoccidioides* genome is not surprising. Coprogen B consists of a fusarinine molecule linked to the dihydroxamate dimerumic acid. In *Paracoccidioides* young cultures the amount of coprogen B in the supernatants is higher than the amount of dimerumic acid while in older cultures this proportion is reversed. This strongly indicates that the dimerumic acid found in *Paracoccidioides* supernatants is a byproduct of coprogen B. Similarly, dimerumic acid was identified in *H. capsulatum* cultures as a breakdown product of coprogen B [61], [62]. Both siderophores were also recognized in supernatants of *Blastomyces dermatitidis* grown under iron-poor conditions [63]. Ferrichrome C and ferricrocin are cyclic hexapeptides in which the acyl group bound to the hydroxyornithine is an acetyl [64]. Ferricrocin is produced intracellularly by *A. fumigatus* and *A. nidulans* for hyphal iron storage and distribution [17],[58],[65]. The basidiomycete *Ustilago maydis* produces ferrichrome and ferrichrome A for iron acquisition [66], while ferrichrome C is produced by the dermatophyte *Trichophyton rubrum*, which also synthesizes ferricrocin [67]. Although both ferrichrome C and ferrricrocin were identified in *Paracoccidioides* cellular extracts, their role in iron storage requires further investigation. Interestingly, the extra- and intracellular siderophores discussed above are not only produced by human pathogens. The plant pathogen *Magnaporthe grisea* and the non-pathogenic model organism *Neurospora crassa* secrete siderophores of the coprogen-type for iron acquisition and use ferricrocin for intracellular iron storage [68], [69]. This demonstrates the broad utilization of siderophores as a strategy for iron acquisition in fungi.

A previous study demonstrated that *P. brasiliensis* plating efficiency is enhanced in presence of coprogen B, dimerumic acid (both isolated from *B. dermatitidis*) and ferrichrome, the latter being the most effective growth factor [70]. Here we showed that ferricrocin and ferrioxamine can also be used by *Paracoccidioides* as iron sources. In fungi, the uptake of siderophore-iron complex is usually mediated by transporters belonging to the UMF/SIT subfamily of the major facilitator superfamily (MFS). The three genes encoding putative siderophore transporter orthologs found at *Paracoccidioides* genome present a MFS1 domain indicating that they belong to the MFS [39]. The presence of more than one putative siderophore transporter in *Paracoccidioides* may reflect its ability to utilize a variety of siderophore as iron sources, including the xenosiderophore ferrioxamine.

Cross-feeding experiments demonstrated that siderophores secreted by *Paracoccidioides* restored the growth of the non-producer *A. nidulans* Δ*sidA* mutant. This strain is unable to grow in standard growth media unless siderophores are supplied [16]. *A. nidulans* encodes 10 putative siderophore transporters [19], [71]. This fact, associated with the lack of a reductive iron assimilation system, is in accordance with the ability of this fungus to utilize xenosiderophores. Beyond the native siderophores ferricrocin and TAFC, *A. nidulans* is also able to utilize iron from enterobactin, a catecholate-type siderophore produced by bacteria, and from ferrioxamine B, a less effective iron source [72]. Thereby, utilization of *Paracoccidioides* siderophores by the *A. nidulans ΔsidA* mutant is in agreement.

Since siderophore production and uptake has been described as an important virulence attribute for pathogens [13], [21], we started checking the influence of these molecules during fungus interaction with murine macrophages. Following inhalation by the host, *Paracoccidioides* propagules bind to macrophages in the lung. Once phagocytosed, fungal cells are able to survive and multiply in non-activated macrophages. However, IFN-γ activated macrophages prevent multiplication of ingested fungus and, consequently, its survival [73]. Here we demonstrated that exposure of *Paracoccidioides* yeasts cells to dimerumic acid and ferrioxamine before co-cultivation with IFN-γ activated macrophages resulted in an increase in survival when compared to cells treated with ammonium ferrous sulfate or BPS. We hypothesize that the siderophore utilization before infection provided the iron requirements for fungal metabolism and for defense against oxidative stress generated by macrophages. Our group previously demonstrated the induction of a peroxisomal catalase (an iron dependent enzyme involved in the detoxification of reactive oxygen species) in *Pb*01 yeast cells derived from infected macrophages [74]. *Paracoccidioides* yeasts exposed to ammonium ferrous sulfate and BPS presented similar survival after co-cultivation with activated macrophages. BPS is a ferrous iron chelator which inhibits reductive iron uptake but does not affect siderophore-mediated iron uptake. Thus, the pre-incubation of yeasts cells in MMcM containing BPS and the additional treatment for 3 h with this iron chelator before macrophage infection probably trigged the induction of siderophore biosynthesis and uptake. This fact may explain yeasts survival after co-cultivation with macrophages. The demand for iron for *P. brasiliensis* survival during interaction with phagocytes was already investigated. Results demonstrated that iron is essential for intracellular transformation of ingested conidia to yeast in murine macrophages [75] and for survival of yeast cells inside human monocytes [59]. In agreement with the growth assay in presence of siderophores, we demonstrated that these molecules play an effective role as iron sources for *Paracoccidioides*. It was formerly demonstrated that addition of FeCl_3_ to *Paracoccidioides* minimal medium is not as effective in the increase of fungus plating efficiency, as supplementation with siderophores [70]. The exposure to ferrichrome also enhanced the survival of the opportunistic fungal pathogen *Candida glabrata* to macrophage killing [76]. Also, *A. fumigatus* employs mainly a siderophore-based iron acquisition system *in vivo* instead of the reductive iron assimilation pathway [13].

Quantification of transcripts level of the putative siderophore biosynthetic gene *sidA* revealed that this gene was induced during co-cultivation of *Paracoccidioides* with macrophages. This suggests that the fungus probably produce siderophores to overcome low iron availability imposed by these activated phagocytic cells. Such strategy is employed by other fugal pathogens. Extra- and intracellular siderophores were shown to be crucial for intracellular growth of *A. fumigatus* within alveolar murine macrophages [77] and expression of siderophore biosynthetic genes was detected during murine infection with conidia [78]. In *H. capsulatum* the expression of the *sidA* ortholog *sid1* was also induced after phagocytosis and required for adequate cellular growth in human macrophages [79].

Altogether, our results revealed the ability of *Paracoccidioides* to synthesize and utilize siderophore as iron sources. Although the production of these iron chelators had been formerly reported, we demonstrated here, for the first time, the identity of the produced siderophores including the intracellular ones, whose production was not mentioned before. Additionally, infection experiments carried out with a murine macrophage cell line revealed that siderophore utilization plays an important role during the interaction of *Paracoccidioides* with mammalian cells. Despite some studies had demonstrated the importance of iron in the scenario of *Paracoccidioides* infection, evaluation of the impact of iron metabolism on fungus pathogenicity was not deeply investigated. This study was the first step of upcoming molecular and functional analysis of siderophore biosynthetic and uptake genes in *Paracoccidioides*. Indeed, studies are being carried out in order to investigate the role of these genes as possible virulence factors in this pathogenic fungus. The added knowledge is clinically important since siderophore biosynthesis and uptake represent possible targets for an antifungal chemotherapy due the absence of these pathways in human cells.

## Supporting Information

Figure S1 **Biosynthetic pathway for fungal hydroxamates.** All the expected genes for siderophore biosynthesis are present in *Paracoccidioides* genomes. NRPSs: non-ribosomal peptide synthetases. Adapted from [51].
(TIF)

Figure S2 **Usptream regions of siderophore genes in** ***Pb*** **01.** Sequences are shown in 5′→ 3′ sense.
(PDF)

Figure S3 **Similarity of** ***A. fumigatus*** **SidI with putative acyl-CoA ligase from** ***Pb*** **01,** ***Pb*** **18 and** ***Pb*** **03.** The amino acid sequences of the orthologs were aligned using the software ClustalX2. Asterisks: amino acid identity. Dots: conserved substitutions. Grey box: PTS2 motif.
(TIF)

Figure S4 **Similarity of** ***A. fumigatus*** **SidH with putative enoyl-CoA hydratase from** ***Pb*** **01,** ***Pb*** **18 and** ***Pb*** **03.** The amino acid sequences of the orthologs were aligned using the software ClustalX2. Asterisks: amino acid identity. Dots: conserved substitutions. Grey box: PTS1 motif. PTS1 scores: *Pb*01 (8.8), *Pb*18 (10.4) and *Pb*03 (10.4).
(TIF)

Figure S5 **Detection of hydroxamate-type siderophores in** ***Pb*** **01 and** ***Pb*** **18 supernatants by the ferric perchlorate assay.** *Pb*01 and *Pb*18 supernatants from three independent cultures in no iron MMcM (Fe -) presented an orange-red color after addition of Fe(ClO_4_)_3_, revealing the presence of hydroxamates. Cultures of both *Paracoccidioides* isolates in the presence of 30 µM ammonium ferrous sulfate (Fe +) were also tested and the change in color was not observed. Sterile MMcM was used as reference (Ref + and Ref -).
(TIF)

Figure S6 **High-resolution mass spectrometry of** ***Paracoccidioides*** **extracellular siderophores.** A: RP-HPLC peak corresponding to coprogen B in Figure 4A was submitted to MS and MS/MS analysis, demonstrating that dimerumic acid is as a breakdown product of coprogen B. B: Longer periods of cultivation result in an increase in the amount of dimerumic acid over coprogen B, as demonstrated by RP-HPLC peaks from *Pb*18 supernatants obtained after 10 days of incubation.
(TIF)

Figure S7 **High-resolution mass spectrometry of** ***Paracoccidioides*** **intracellular siderophores.** RP-HPLC peaks displayed at Figure 4B were submitted to mass spectrometry analysis for molecular masses definition of ferricrocin (A) and ferrichrome C (B). The four different ionizing adducts are shown.
(TIF)

Figure S8 **MS/MS fragmentation analysis of ferricrocin and ferrichrome C.** Fragments with identical molecular masses (m/z = 370.094, m/z = 398.089 and m/z = 455.111) are framed in red. Fragments that show a molecular mass difference of 15.99, which corresponds to the mass difference of the two siderophores, matching the mass difference of serine (in ferricrocin) and alanine (in ferrichrome C) are framed in blue (m/z = 498.153 for ferrichrome C plus m/z = 15.99 is m/z = 514.145 for ferricrocin; m/z = 583.169 for ferrichrome C plus m/z = 15.99 is m/z = 599.159 for ferricrocin; m/z = 737.244 for ferrichrome C plus m/z = 15.99 is m/z = 753.234 for ferricrocin).
(TIF)

Table S1 **Oligonucleotides primers used in quantitative RT-PCR.**
(DOCX)

Table S2 **Accession numbers of** ***Paracoccidioides*** **siderophore genes available at** http://www.broadinstitute.org/annotation/genome/paracoccidioides_brasiliensis/MultiHome.html **.**
(DOCX)
