# Supplementary figures and images for: Hydroxamate Production as a High Affinity Iron Acquisition Mechanism in Paracoccidioides Spp
Source: PLoS One. 2014 Aug 26;9(8):e105805. doi: 10.1371/journal.pone.0105805 (PMC4144954; doi:10.1371/journal.pone.0105805)

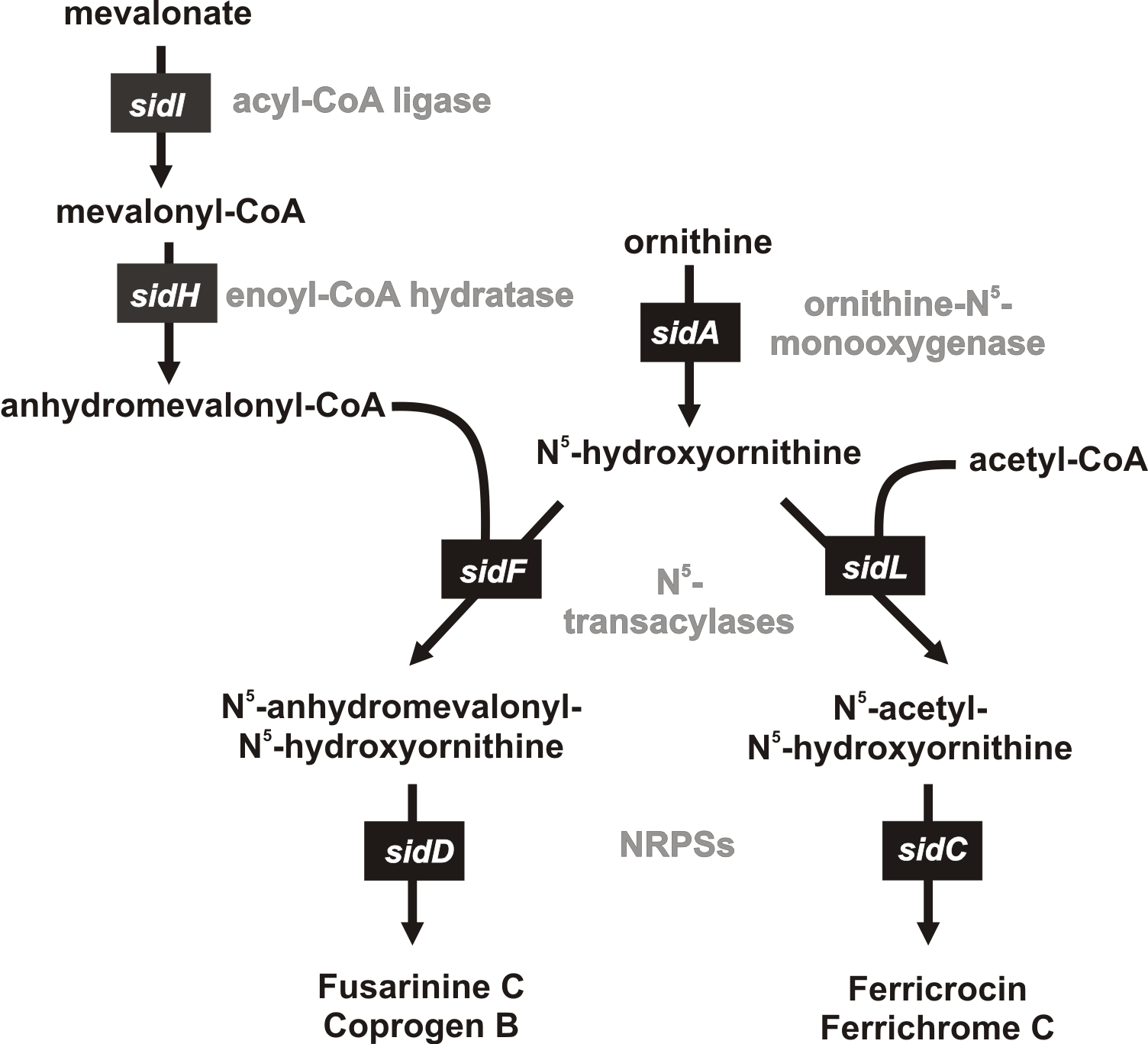

Supplement: Figure S1 — Biosynthetic pathway for fungal hydroxamates. All the expected genes for siderophore biosynthesis are present in Paracoccidioides genomes. NRPSs: non-ribosomal peptide synthetases. Adapted from [51]. (TIF) [file pone.0105805.s001.tif]

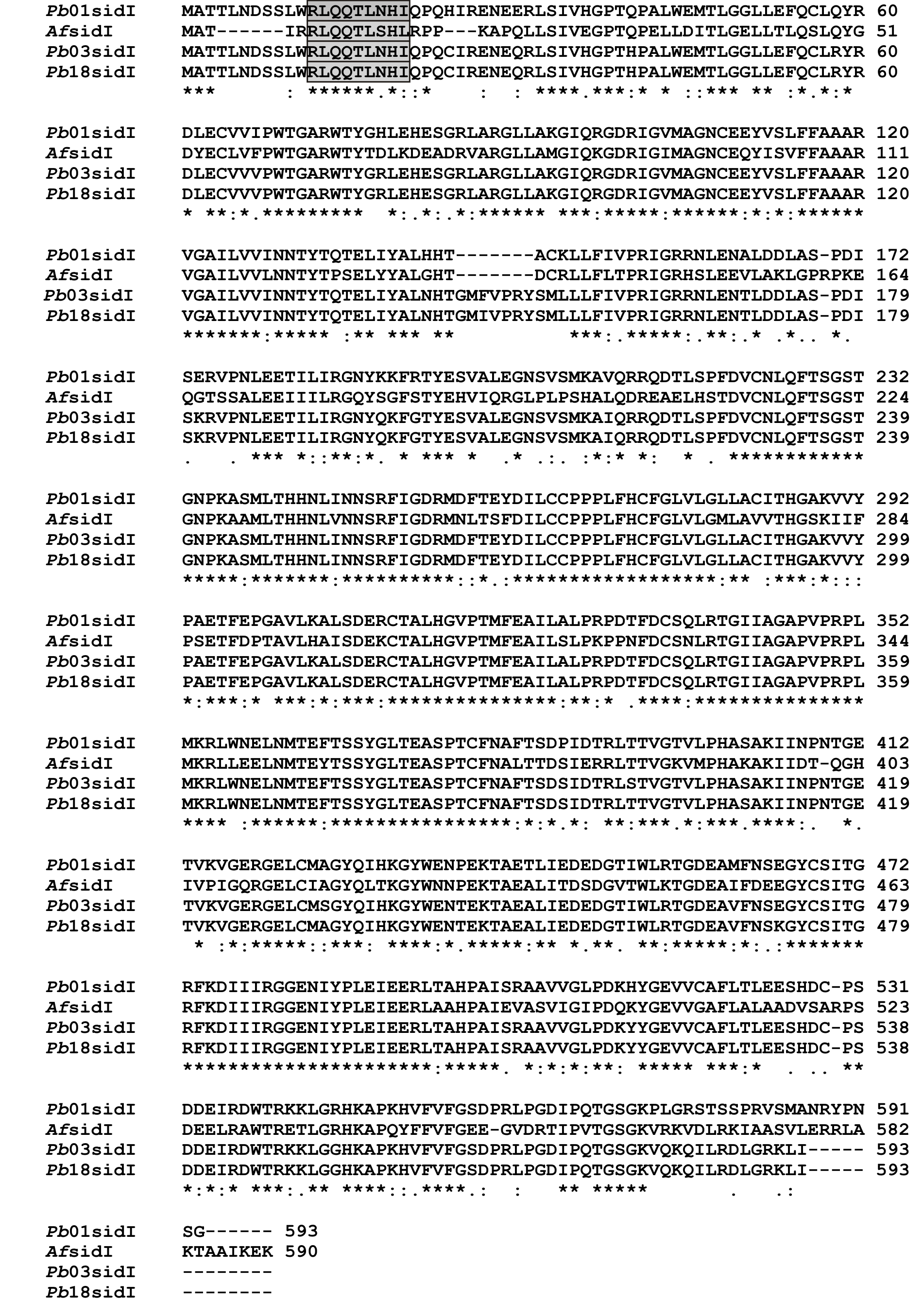

Supplement: Figure S3 — Similarity of A. fumigatus SidI with putative acyl-CoA ligase from Pb 01, Pb 18 and Pb 03. The amino acid sequences of the orthologs were aligned using the software ClustalX2. Asterisks: amino acid identity. Dots: conserved substitutions. Grey box: PTS2 motif. (TIF) [file pone.0105805.s003.tif]

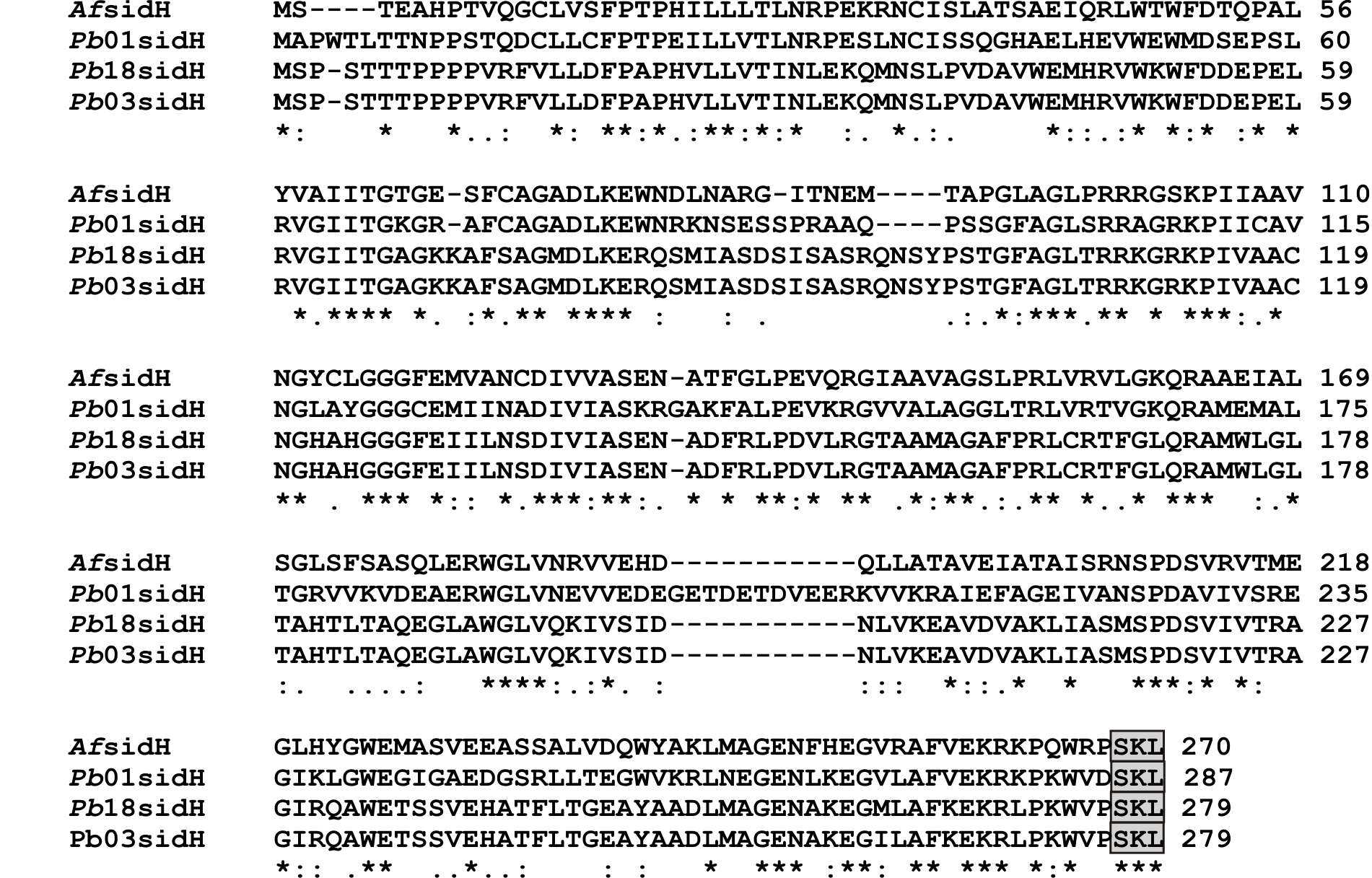

Supplement: Figure S4 — Similarity of A. fumigatus SidH with putative enoyl-CoA hydratase from Pb 01, Pb 18 and Pb 03. The amino acid sequences of the orthologs were aligned using the software ClustalX2. Asterisks: amino acid identity. Dots: conserved substitutions. Grey box: PTS1 motif. PTS1 scores: Pb01 (8.8), Pb18 (10.4) and Pb03 (10.4). (TIF) [file pone.0105805.s004.tif]

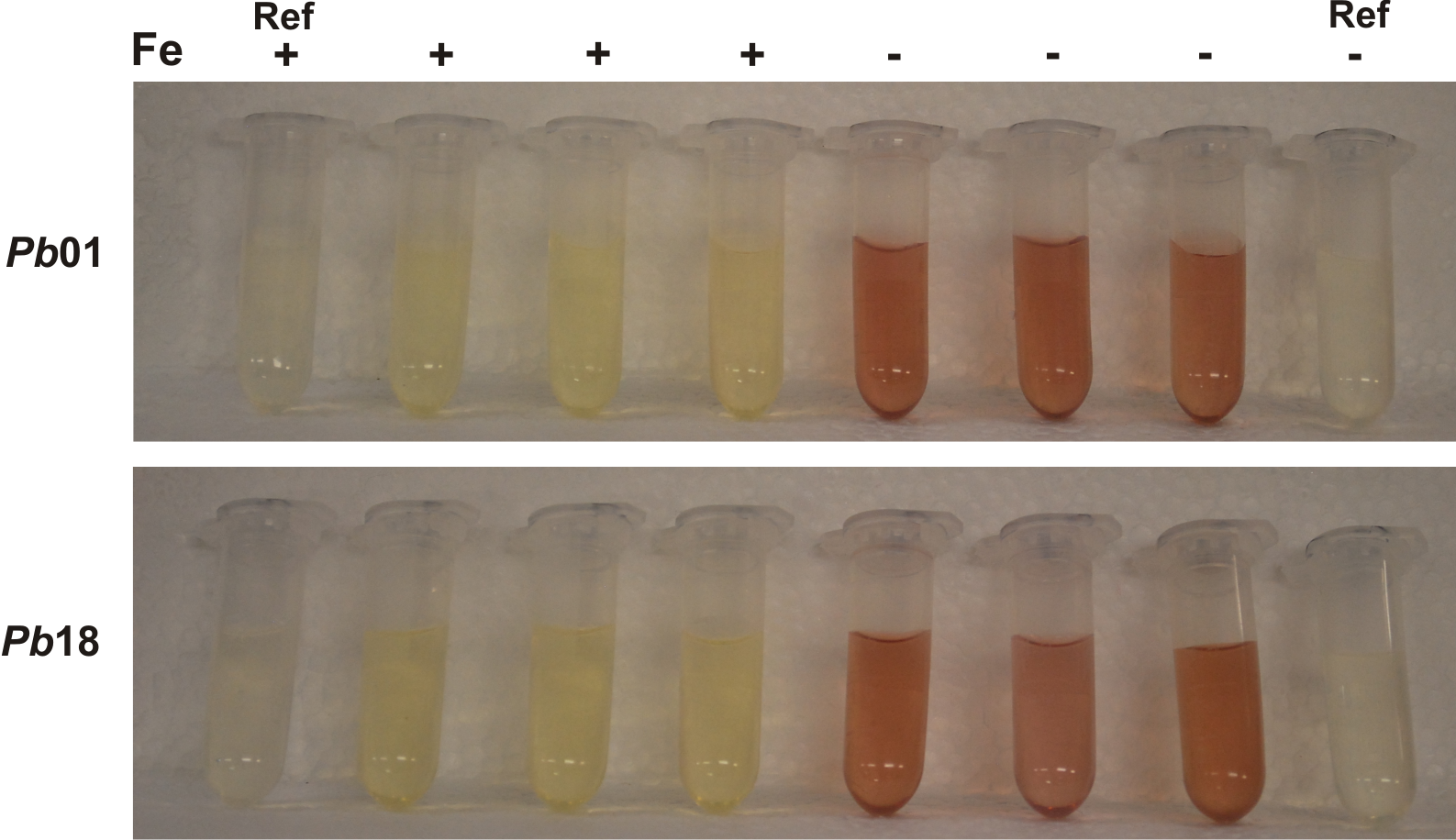

Supplement: Figure S5 — Detection of hydroxamate-type siderophores in Pb 01 and Pb 18 supernatants by the ferric perchlorate assay. Pb01 and Pb18 supernatants from three independent cultures in no iron MMcM (Fe -) presented an orange-red color after addition of Fe(ClO4)3, revealing the presence of hydroxamates. Cultures of both Paracoccidioides isolates in the presence of 30 µM ammonium ferrous sulfate (Fe +) were also tested and the change in color was not observed. Sterile MMcM was used as reference (Ref + and Ref -). (TIF) [file pone.0105805.s005.tif]

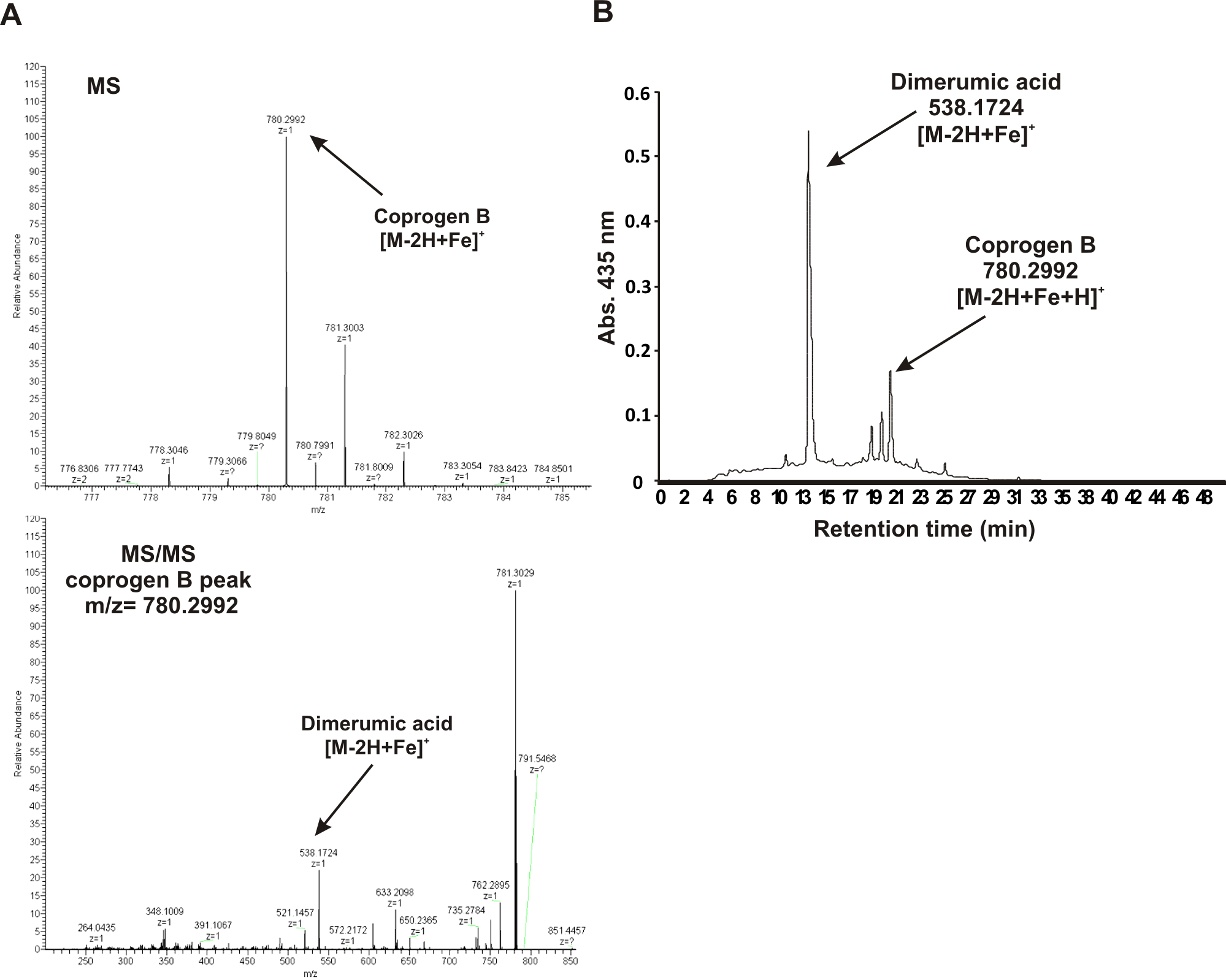

Supplement: Figure S6 — High-resolution mass spectrometry of Paracoccidioides extracellular siderophores. A: RP-HPLC peak corresponding to coprogen B in Figure 4A was submitted to MS and MS/MS analysis, demonstrating that dimerumic acid is as a breakdown product of coprogen B. B: Longer periods of cultivation result in an increase in the amount of dimerumic acid over coprogen B, as demonstrated by RP-HPLC peaks from Pb18 supernatants obtained after 10 days of incubation. (TIF) [file pone.0105805.s006.tif]

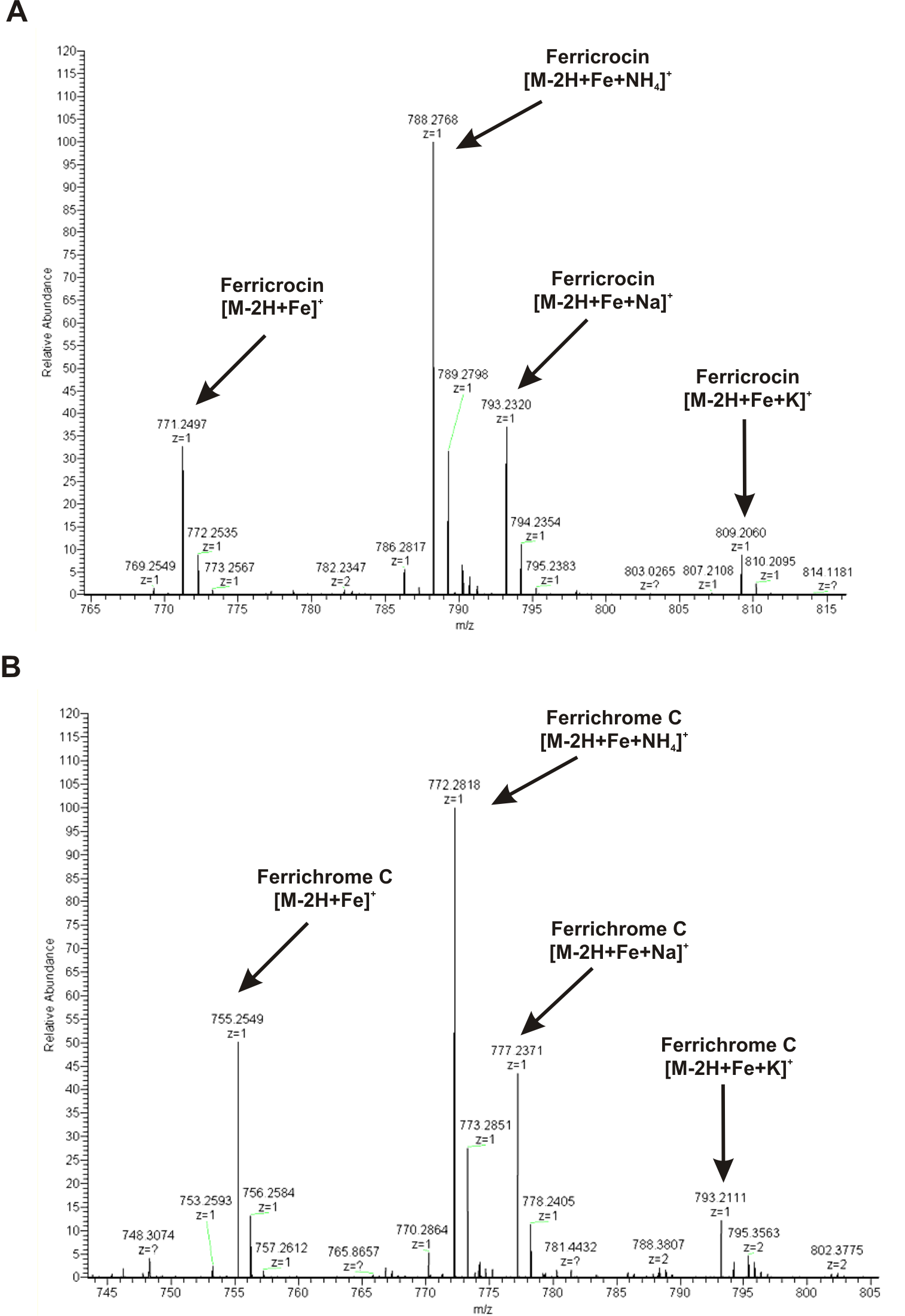

Supplement: Figure S7 — High-resolution mass spectrometry of Paracoccidioides intracellular siderophores. RP-HPLC peaks displayed at Figure 4B were submitted to mass spectrometry analysis for molecular masses definition of ferricrocin (A) and ferrichrome C (B). The four different ionizing adducts are shown. (TIF) [file pone.0105805.s007.tif]

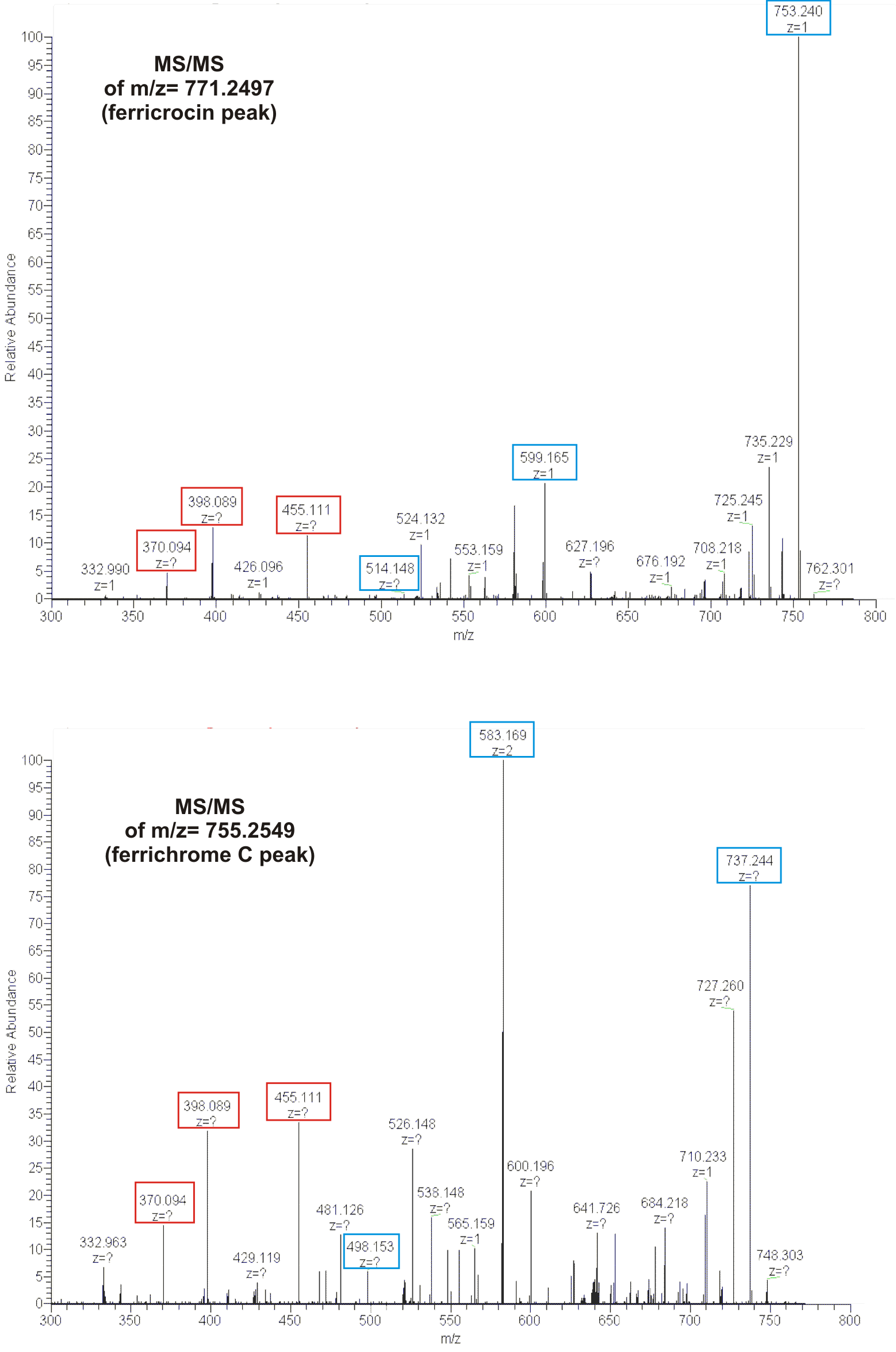

Supplement: Figure S8 — MS/MS fragmentation analysis of ferricrocin and ferrichrome C. Fragments with identical molecular masses (m/z = 370.094, m/z = 398.089 and m/z = 455.111) are framed in red. Fragments that show a molecular mass difference of 15.99, which corresponds to the mass difference of the two siderophores, matching the mass difference of serine (in ferricrocin) and alanine (in ferrichrome C) are framed in blue (m/z = 498.153 for ferrichrome C plus m/z = 15.99 is m/z = 514.145 for ferricrocin; m/z = 583.169 for ferrichrome C plus m/z = 15.99 is m/z = 599.159 for ferricrocin; m/z = 737.244 for ferrichrome C plus m/z = 15.99 is m/z = 753.234 for ferricrocin). (TIF) [file pone.0105805.s008.tif]
