# Supplementary material for: Hydroxamate Production as a High Affinity Iron Acquisition Mechanism in Paracoccidioides Spp
Source: PLoS One. 2014 Aug 26;9(8):e105805. doi: 10.1371/journal.pone.0105805 (PMC4144954; doi:10.1371/journal.pone.0105805)
Supplement: Figure S2 — Usptream regions of siderophore genes in Pb 01. Sequences are shown in 5′→ 3′ sense. (PDF) [file pone.0105805.s002.pdf]

Upstream region of *sidD*

HGATAR: -215; -312
ATG: *sidD* start codon

TGGATGGGGTAGGTTTTACGTTCCATTCTCGGCCCTTACGCGAACGACCATGAGTATGTTCGAC
TATCTGTGTGGGTAAGTCCTTAGCCGCTATTTACCGCTGGTCTCCATTATCTGCCTTAGGGCGC
TCGGTGCAGGTCGTTTTGCAAAAATCGATCCTCGCAAAAAATGAAATTGAAAAATGAAGGTCAG
TAGTCCATTACGCGATATGGACGGTTTTGGAGTAAAGTTTCCATCCTTCACTTCACAAGCCAAG
GGTACCCTGGTAGGAACGTATGACGCATTGCACAATATATCCTGACGACAGGCCTGAGGTGACG
AACCAGCGTAAGGATACATCAATTAAGGTGGAACTTGGTTGCAATTG**C**GATA**A**AATACCAGGTG
GCAAAATATAAACTGCTAGGCGTAGGGGAGTGTTTGGTAAAGAGAAATCGCCCTGTTCAGACTT
TGTATATACGATGGGA**T**GATA**A**AAGTCATCACATATTCTTCAAACAAGGCACCAGCAAGGACAA
CATCTAGCTGTCAACTTCAGGAATCAATTGTACTCGATTGCACCATGAACAGCAATCTATCCGC
ATGTGAAGATGTCAAAACAAGCGGAGGCGTTCACTTAAACGGTGATGCAAATGGTAGGGTAGA
TGCCATCTCGATCGAGCAACCACTTTTTTTAACCGGAACATG

Upstream region of *sidF*

HGATAR: -844; -1821; -1985
RATCWGATAA: -688; -1329
ATG: *sidF* start codon

AGATGGATGGTGAACTCTTTCGGGACTTGATCGAGCTGAATTTCATCACTGACAAGAAACGGTA
TTGCCAAATGTCAAGGTGATACAATGCGCAGGGCAAACTGTGGCATTATTTCAGCGGCCACAGA
GGAGGAAATTGTGGGATGGGGATTTCTCTTTTCATGTCTGTTTGAGAACGTTTTACGATGCTTT
TTTCAAGTCTTTGCAAAAACAGGGATGGTATGAAAAGTGAGTGAGATGAGGAAATAAGAGTTTG
AATTAACTTGATGCATGGAAGCTCTTGCTTTACTCAATAAAGATGCAGATATAATATTGATATT
CAATAATTTGACAATGGGCTCAGAAGTGCCCTTCTAAGCTTCGCATTCTGACAGCGGAGTTGAC
CTGGTCTACTCATACGTGGAGTTAGTTATATAACTTCACCAGATAAGGCGTGTGGATCAAATCA
ATACTCTTCAACTTGTACTATTTCCACCTGGACGCATAACTACTGACTGGGAAAGGTTTGAGCA
GTGGCACTTGATCTATTCAGCCAATTTGACAGAGAAAACTTAGGTATATAAATTATCTTTTTAA
TTTGTCAAAACATGATAGCCACAAAACATGTCCCTTTCCTTGTCCATAGAACATTCATGCCCTC
ATCGATTCCTTTCCCCTCATTCTCTTCTCTGCACCCCCCTGTTATCACATCCCTTCATCCATGG
AAATTTCTATTTTCAATCTACGAAACCTCCCGAGGATAGATGATGCACGGTAACGTAATTTTCC
ACAAAAAAGGGAACTGTGTAAATTTCTTCCGAAGTTAAATCATGTAAACCCGANNNNNNNNNNN
NNNNNNNNNNNNNNNNNNNNNNNNNNNNNNNNNNNNNNNNNNNNNNNNNNNNNNNNNNNNNNNN
NNNNNNNNNNNNNNNNNNNNNNNNNNNNNNNNNNNNNNNNNNNNNNNNNNNNNNNNNNNNNNNN
NNNNNNNNNNNNNNNNNNNNNNNNNNNNNNNNNNNNNNNNNNNNNNNNNNNNNNNNNTCAAGAG
GCTGTCTTTACATAGGATGAACTAAATTGGCGCCAAACGTGTCCACGCTAGAGCTT**G**ATC**A**GAT
AATGAAACTTCTGCAAGCAAATGTAGAAATAATTTCTTTTCTTCGTTTTGTGTAGCTCTACTAA
AAAGTTATTCGCGATTGTACCGTCTTTTCCCATTGTCAGAGCTCTTACTATTCCTGCATGGTTT
TGACACGGAAACACTTTGGTTGGTTCACTGAAGCGGTGTGTGGTCCTAGAACTCCGAACAACGG
AGAGCTTTGACCCAAATCCTGCACATAGCCTTGAAAACTAGGGTCCTCATATTATTCTGCTTGT
CACGTTACATGGTCCTCGGGGAGGGAGGATGGCACCTTTGCATTCCTGGTGTTACATTACTTGG
AGCGGGGACACTCGGAATTCCATCAAATTCTTTGCAATTGCCTTTGCTCACCCCTGACAATTGC
CGGCGTGATCCGGGAAGAACGACGGAGAGGCCTTCTCGAATAGTCTCCCTATTCCATGTCCTTG
TCATCCGCGTGGTATACCCGGAGAAATGTTGATAACTGGTAGACAATAACAATACATTTTGAGG
TTTTCAAGTACCTTGGTTGAACCCACAGCAGAGATTCTTCAGTGGTTTCTTGACAACCGGGTCA
AATCGGGATGTTTGTCATGTTGACCACCCCACCTGTCGGCTTGTTCGTGTGTTAAAC**G**ATC**T**GA

TAATGCATGCGGAGCGGCAAAGTAATGGCCTCAAGCAAACTTAACAAGCAGCGAAGAAAGGAGA
ATCTCGCTAATCATTCGTTGGTGTTTTTCTCCGGGTTGAGTTTATTGATTTGTTATCTTATCTG
ATGCGCTTTGACCTGGGTTGAAATAATATGACTAAAAATACCGACTATCAGATGATCTGATTAG
ATATCCACGATTTGTAACGCCAGTTATGGAGTGCAACTGACCGTAATAGAGTTATACTCGTATT
GACAGGTTAGCGAAAATACTGTACTATAGATCGCAATTTTTCACGCACGGTATTATAAACATGA
CCCTGGCCTTATGGAAACTTGTATTTTCCGCCCGGGGAGGCATATTATGTATGGACGCAATACA
ATAGCCAGGTGAAGTCCAAATTTCCGGGCATTCGTGGATATGAACCGAGGAGTGAAGAAGCACA
CTCATAACGGTTTCTTCTATCCGGGTCGATGGAGTGGAGGCCGCCTGAAGTATTAAGGTGTCAA
CACATGGATCGTGGATTGGTTCTTGCAAAGGAGAGCATTGAAGAATTTTTCCTTATTGGATCCA
TCTTCTCTAATCGACTCACTGACCTTTTATATTTTTGAATAACTTCGGAACTTACCTCTTCTCC
CTTTCGTATTCTGCCGTCTATTCGCAGCTGCATACTGTACAATG

Intergenic region between *sidA* and *sidI*

HGATAR: -640
RATCWGATAA: -623
ATG: *sidA* start codon

TATAGCAATTGCGTTTTTGTGTTTATTATACCCTTACACACCCCGAAGGCCCAATGTTGCATTT
CTTTACAACAGCTTTTACATATAGTTTTTTTATTTATTCTTCATTTTTTCCCCCCTTCCTCCTT
AAGGATTTGCCGCAGCCCGTAAGAGGTAATGCACCCCCTGTCAAGTCCATCTTGTCTAAGTCTC
CCTTACCAGGGTTGATGGATTTGAAACATGCAAAAAAGAGAAGTTGATGGACTACTGTTCCAAT
GTCTCTAGCGTACTAAGCGTATGAAATGTTACGGTTGTGGGGGCATGTGGCAGTGCAGAGACTC
TTCCATCCGTCGCGGATTCTTTATTACAGACAACACGAAAACGAG**A**GATA**A**GGAATCTAATC**A**A
TC**A**GATAATCTTACCAAACCCGTAATTTCGAGACAAAGTTACACTAGAGTGTCAGGGACAAAAA
TACACAAAAACAGCGATACATCGGTTGTATTTCCGGCTGCAAACGGAGTCCAGTTCTTCGGACC
TCAGGAACTTTCACCCGGAGTTTTTCCCTTCTCAACGGACTCTCTCTCTTGCAAAAACGGCAGC
TATTTTCTTTCCTTGGCCAGCCACAACAAGGCTTGTTGGCTTGTTAGACACCCAATCCGCCATC
GTCTGAGATTTCGGCTTTTTGGAGAATACGGCCATAGTTGCTATATTCTGCTCTAACAATTTTG
CAAATTGCGGTTTTACGTTACCGTCGCATCCTTGACAGGGCACGCGAGGTTCAACACTGTGATT
GATGCATGGTATATGACACTCCAACAACATCAGGCGATGCCAATCGCCGGACCTAACCAGCAGT
GTGTTTTTGCTTCAGCTAAGGGTTCTTAAGTTTTCGAATTCCCTCTAATTTCCCACCGGCAACG
TTCGATTATTTGTTACGTTGACATCATTTTTTTTAATCACCTATTTTATTTACGCTGCTGAGAT
TACCTCGTACTATCCCTCTCACCATATTCACTACCATCATTCCCCATG

Upstream region of *mirB*

HGATAR: -1782; -2164; -2397; -2616; -2958; -2969; -3273
RATCWGATAA: -1946
ATG: *mirB* start codon

CTACAAGGCAAAGGCAAACCTTTCTTGAAAAGATGAGATTCTCCAGAATGTAACACAAAGTACG
AGTTGTATGTACAATGCTTTGATCCAAGGAACAAAATGATGATAGTGTATTATTAAACCCCCTA
TTTGCGAGAGAAACTCATAGGTTGTCAAAAAATACCCACAATGGGTTAGTTGAACTTCCGTCTG
CTAAGTTATGTCTGGCAAAGTGAAGTCAAGCACATGGGAAAATGAGAACAACTGTAAATACTTT
TCTTCTAGACATTATAATTGCTTTTTGCGTCTGCAACGTAGCTTCTAATCCCAAACGTTTTTGC
AATGGGTAGCTGGGCGAAGTAATATAGGACACCAAAGGAAACATGGTAGCTACGTGCTGGTTTC
CACCAGCGTAGTAGATATACCCTCGATAAGCACCCGATAATCAAAGAAAAAACTAACGTGCTAC
AACTTTACTATTTCTTAACCTTTAAGCTTTCTAGGCCGACCTTTTAACCCCTTTCCTCTTCTTC

AGGTAACAACTGTTCTCACCTCATTACTCAGAGTGTACCAGAAAAATACCGAGACCGTCGCTAC
CAGTACGGGGACGCTTTCATCGTAGGGATGTGGTCATCAGTGATGGCTCCATTCCAAACTTCCG
CAACCTCTTTTCCACTCAACTGATCTGCAAGCCCGGCGGATGCATAGTGGAAGTTAAATGCTGA
CTCTGTAGCACGGAATGAAATCTTGAGCATTTCGCAAATCAAGCTTGTCACTGCACCGATAGAT
GATGATCATGCTTGAAGGTTCTCACATCGTTCATACAATATCTCGAATCATCATAGGAGATTAA
AAGGGAAGAGCAAAGAACCAGAGGTTAGCTGACAGTGCTGGCGTTGGAGCTGAAGTGACTGAAG
TTGTTGAAGTAGTGGGAGACGACATCTCAATAATTTCAGAGCTGGCTAGATGTGATGTTGCACT
TTCAGGAGCAGAGGTGACTTGATAGGCTGTTTTAGGGGCACAGGTGGCTGGATAGGAGTTTGCG
CTGCCTTCAGTGGCAGAGGTTGGTTGCAGGAGTGGCTGCGCTGCTCTCGGATATACTCTCAGTG
GCTGAGGTGGATGTTTCAGGGGTTGGGGTCACTGGAGAGGACGTCTCAGTAGTTGAAATGATGG
GAAGGCTGTTAAAGTGGTCGAAGTGGTGGGGGAGGACATCTCAGTAGTTAAAGTGACGGAAGAT
AGGTCATTGAAGGGGTCAAGGTGAATGTTTCGGGGTTGAAGTCACCAGAGAAAATGTATCCGTG
GTTGAAGAAAAAACCTCCCTTTGAGATATGTAAGTCCAACCACCTTTAGTTTTCATCGCATATA
TCAACAAATACCTCGGTAGGTTGACTGGGGCCCAAATACGTCAAACCTCTGGAAGGCTTCTTGC
GCCTTGCCTTGTGCAGGAACAT**G**ATC**A**GATAAATATACGTAACCACATAACCGACACACGAAAA
ATCCTCCCTGGACTGGAAAGGCATGAAACCTCAATGGATGAGATCACATCCTTCTCCTCCTCAT
TGGACTCATCCAGTCTAACGAATTCAATAGTGTTTGTGCCATTATATGTGCACGCAGAAGATAA
ACGCAGCTTACACGGGTGTGCCGAGCACAATTCAACTGGAAACGCATCAACCAGGTACAACCCT
AAGTGATGATGGAAGAAGTTGGAAGAAACATATTGGCACCCAACTGGCTGAATTCGTAAAGTAG
GTTCCATGAAGTGATTTGGGAACTTTTGTGGTTTTTGGAGGTTTGTTGAATTCTGAATGCCTCA
TTGTCGGGGTCGCTGGAAACGCGGTCCACACTGACAGACTGTCCATGTTCCAAACAAACAGTTG
GCCGGTCTTCCAAGGATGAGAAGAAACCTTGGTGTCTGAACAAAACTGACTTGATGCCTTTGAA
ACTAGCGTTCAAGGAAGGAATCCCGACATACCCCAGAACATTTCATCCGTCAAATACCTGACAA
AAGAACACAAAGGCGGAAATTTCTGTGGTTATAAAAAATAATAAAGAATGAGCTTTGACTATGA
ACGGACGAGGATGGTTGGAACGTATTGTAGACCGCAACCACTGTGTCCTTTGATGATCTGCCTG
CTCTGTGGGTAAACTTAAAGTCACATCTCCGATATTCGTGCTCCAGTTTATTAATATCATCCTC
GGGAGCGTGGGCCGTGGACAGAGCAGCTCGTTCCACTTACCGTCAATACCCATTAACAAAGATT
GATCATTGCAACATCCGCACTCTGGTGCCTCTTCCAGGATCACTGCTGATTTTGAAATGTGCTC
TTTCGTCCTGCCCTTACAAAACTGCGTCATGTAAAGTGGTCTTTGCATCCATGCTTTCGGCTAC
TTAGGAAACCCACCTGTAGTTGTCGAAAGGTTGATATCAGATGAGAATAACACAACAGGAGAGG
AGCCCTACCTATAATCAGTCCTAACATATGGCTCATGGAAACACTCATATAACTGAAAGATTCT
CACGAAAGTTGGTACAGTGACCTCCATGTGACGACTTGTCATTTCCAAATCTAAAGAACGAGGT
TTCTCTACTGTCAAACAACTAGCTACATCATTGAGAAGGCGATCTCCGTAATCATAGGGCACTG
TGCAACGCAAGTTCCATATGGCACTATGCCCCACTCCGGAAGACTTTATTTGAACATGTTGCAG
GCTGTGATGGAGTCCGAATCGATATCATGGAAGGTACCTTAGTAGAACTGGCGGTGTCGTCAAA
TGTCCAGCGCCGGGTCTCTATCACGTGAGAGAACCGCCGTGCCCGTAGAATACGGCACAAACGC
TGAGTTCTGATTTTGCACGACCATCCACTTTTACCTCAAGTTCCCCAGACGCTTCAATCCATCT
TGGAACCACTCCTACATCTTCCACACCTGAGCCTCCCGTGGGCCTCCATAACTCCATTCTCAAA
ATGGCAAATCTGGATTTCAAACCCCATTCTCCGTATTACGAGGGACGCTACAACGTACGGTACC
GTATAGACAAGTAATACAGTATAGGTATAGTACATGTATTATACCGTATAAGAACGTATTTACC
TCTAATCTTATCAATCGGTCTTATCAGATGGGATCCTAGTGCCGCATATCCTTGTACATGTATG
GTATTGTCACAACAGAGAATTACATCGCACTTCCCGGCCAGAAAAAGCAGCCAGAAACACAAAC
CAGGATCACAGCTCACTCACGTCACTGGCGGACACTGCTCCGCACCTAAGGAAATCTCTCTTTT
GGAGAAGTCATATTAAAATGGCCTTGCCACGCCAGTTGCTCTCCTTTTCGTGTTTGAAGTCTTT
GTTCCACCTCTACCCGACTTCCCAGCCCGATTTCTCACTCCCCTCACCATG
