# Supplementary material for: Hydroxamate Production as a High Affinity Iron Acquisition Mechanism in Paracoccidioides Spp
Source: PLoS One. 2014 Aug 26;9(8):e105805. doi: 10.1371/journal.pone.0105805 (PMC4144954; doi:10.1371/journal.pone.0105805)
Supplement: Table S1 — Oligonucleotides primers used in quantitative RT-PCR. (DOCX) [file pone.0105805.s009.docx]

| **Gene name** | **Forward primer (5´→3´)** | **Reverse primer (5´→3´)** | **Amplified product (bp)** |
| --- | --- | --- | --- |
| *sidF* | GAAATCTATTGGGCCAAGGAG | GTTCGTCGAGGAAAATGTAATG | 160 |
| *sidC* | TGGTGGTGAGCAAGTGGGG | CGTTGGCCCCGTAGTTTAAC | 180 |
| *sidD* | CGGAAAAGTATGGCGATCTAG | GCATAGCCTGAGACACTGTC | 186 |
| *sidI* | CAATGCTGACACATCACAACC | TAAACCACCTTCGCCCCATG | 163 |
| *sidH* | CTGGGGCAGATTTGAAAGAATG | TGGCAATGACGATATCGGCG | 186 |
| *mirB* | GTCTTCTACTGGGTCGGGTAT | GACCATTCAGGAAGGCTGTC | 157 |
| *mirC* | CAGAATGTGGTGAACGCCGT | AGAATTTGCAGTCCTGTTGAAC | 194 |
| *α-tubulin* | ACAGTGCTTGGGAACTATACC | GGGACATATTTGCCACTGCC | 136 |

**Table S1.** Oligonucleotides primers used in quantitative RT-PCR.
