# Supplementary material for: Hydroxamate Production as a High Affinity Iron Acquisition Mechanism in Paracoccidioides Spp
Source: PLoS One. 2014 Aug 26;9(8):e105805. doi: 10.1371/journal.pone.0105805 (PMC4144954; doi:10.1371/journal.pone.0105805)
Supplement: Table S2 — Accession numbers of Paracoccidioides siderophore genes available at http://www.broadinstitute.org/annotation/genome/paracoccidioides_brasiliensis/MultiHome.html . (DOCX) [file pone.0105805.s010.docx]

**Table S2.** Accession numbers of *Paracoccidioides* siderophore genes available at http://www.broadinstitute.org/annotation/genome/paracoccidioides_brasiliensis/MultiHome.html.

|  | ***Paracoccidioides* accession number/**  ***A. fumigatus* similarity** | | |
| --- | --- | --- | --- |
| **Gene** | ***Pb*01** | ***Pb*18** | ***Pb*03** |
| L-ornithine 5-monooxygenase (*sidA*) | PAAG_01682/  63% | PADG_00097/  64% | PABG_03730/  64% |
| Acetylase (*sidF*) | PAAG_01680/  63% | PADG_00100/  63% | PABG_03728/  63% |
| Succinylbenzoate-CoA ligase (*sidI*) | PAAG_01681/  79% | PADG_00099/  77% | PABG_03729/  77% |
| Carnitinyl-CoA dehydratase (*sidH*) | PAAG_06469/  63% | PADG_01543/  63% | PABG_03016/  62% |
| D-alanine-poly(phosphoribitol) ligase subunit 1 (*sidC*) | PAAG_08527/  44% | PADG_05295/  45% | PABG_04670/  45% |
| D-alanine-poly(phosphoribitol) ligase subunit 1 (*sidD*) | PAAG_01679/  61% | PADG_00102/  61% | PABG_03726/  61% |
| Siderophore iron transporter (*mirB*) | PAAG_01685/  60% | PADG_00095/  61% | PABG_03732/  61% |
| Siderophore iron transporter (*mirC*) | PAAG_02233/  78% | PADG_00462/  54% | PABG_04747/  79% |
